# Outcome assessment for out-of-hospital cardiac arrest patients in Singapore and Japan with initial shockable rhythm

**DOI:** 10.1186/s13054-023-04636-x

**Published:** 2023-09-12

**Authors:** Yohei Okada, Nur Shahidah, Yih Yng Ng, Michael Y. C. Chia, Han Nee Gan, Benjamin S. H. Leong, Desmond R. Mao, Wei Ming Ng, Taro Irisawa, Tomoki Yamada, Tetsuro Nishimura, Takeyuki Kiguchi, Masafumi Kishimoto, Tasuku Matsuyama, Norihiro Nishioka, Kosuke Kiyohara, Tetsuhisa Kitamura, Taku Iwami, Marcus Eng Hock Ong

**Affiliations:** 1 Health Services and Systems Research, Duke-NUS Medical School, Singapore, Singapore; 2 Department of Preventive Services, Graduate School of Medicine, Kyoto University, Kyoto, Japan; 3 Department of Emergency Medicine, Singapore General Hospital, Singapore, Singapore; 4 Lee Kong Chian School of Medicine, Nanyang Technological University, Singapore, Singapore; 5 Department of Preventive and Population Medicine, Tan Tock Seng Hospital, Singapore, Singapore; 6 Emergency Department, Tan Tock Seng Hospital, Singapore, Singapore; 7 Accident & Emergency, Changi General Hospital, Singapore, Singapore; 8 Emergency Medicine Department, National University Hospital, Singapore, Singapore; 9 Department of Acute and Emergency Care, Khoo Teck Puat Hospital, Singapore, Singapore; 10 Emergency Medicine Department, Ng Teng Fong General Hospital, Singapore, Singapore; 11 Department of Traumatology and Acute Critical Medicine, Osaka University Graduate School of Medicine, Suita, Japan; 12 Emergency and Critical Care Medical Center, Osaka Police Hospital, Osaka, Japan; 13 Department of Critical Care Medicine, Osaka Metropolitan University, Osaka, Japan; 14 Critical Care and Trauma Center, Osaka General Medical Center, Osaka, Japan; 15 Osaka Prefectural Nakakawachi Medical Center of Acute Medicine, Higashi-Osaka, Japan; 16 Department of Emergency Medicine, Kyoto Prefectural University of Medicine, Kyoto, Japan; 17 Department of Food Science Faculty of Home Economics, Otsuma Women’s University, Tokyo, Japan; 18 Division of Environmental Medicine and Population Sciences, Department of Social and Environmental Medicine, Graduate School of Medicine, Osaka University, Osaka, Japan

## Abstract

**Background:**

Singapore and Osaka in Japan have comparable population sizes and prehospital management; however, the frequency of ECPR differs greatly for out-of-hospital cardiac arrest (OHCA) patients with initial shockable rhythm. Given this disparity, we hypothesized that the outcomes among the OHCA patients with initial shockable rhythm in Singapore were different from those in Osaka. The aim of this study was to evaluate the outcomes of OHCA patients with initial shockable rhythm in Singapore compared to the expected outcomes derived from Osaka data using machine learning-based prediction models.

**Methods:**

This was a secondary analysis of two OHCA databases: the Singapore PAROS database (SG-PAROS) and the Osaka-CRITICAL database from Osaka, Japan. This study included adult (18–74 years) OHCA patients with initial shockable rhythm. A machine learning-based prediction model was derived and validated using data from the Osaka-CRITICAL database (derivation data 2012–2017, validation data 2018–2019), and applied to the SG-PAROS database (2010–2016 data), to predict the risk-adjusted probability of favorable neurological outcomes. The observed and expected outcomes were compared using the observed–expected ratio (OE ratio) with 95% confidence intervals (CI).

**Results:**

From the SG-PAROS database, 1,789 patients were included in the analysis. For OHCA patients who achieved return of spontaneous circulation (ROSC) on hospital arrival, the observed favorable neurological outcome was at the same level as expected (OE ratio: 0.905 [95%CI: 0.784–1.036]). On the other hand, for those who had continued cardiac arrest on hospital arrival, the outcomes were lower than expected (shockable rhythm on hospital arrival, OE ratio: 0.369 [95%CI: 0.258–0.499], and nonshockable rhythm, OE ratio: 0.137 [95%CI: 0.065–0.235]).

**Conclusion:**

This observational study found that the outcomes for patients with initial shockable rhythm but who did not obtain ROSC on hospital arrival in Singapore were lower than expected from Osaka. We hypothesize this is mainly due to differences in the use of ECPR.

**Supplementary Information:**

The online version contains supplementary material available at 10.1186/s13054-023-04636-x.

## Introduction

Out-of-hospital cardiac arrest (OHCA) is a fatal condition with high mortality, which can occur in anyone and at any time. The annual incidence of OHCA is between 40 and 170 per 100,000 inhabitants in Asian and European countries and cardiac arrests kill more people yearly than cancer, stroke, or trauma [1–4] . To save patients with OHCA, a coordinated set of actions is required, known as “the chain of survival” [5, 6] . This includes immediate recognition of cardiac arrest and activation of the emergency response system, early cardiopulmonary resuscitation (CPR), rapid defibrillation, effective advanced life support, and post-arrest care [5–8].

Despite the best efforts of conventional resuscitation, some OHCA patients do not recover their own spontaneous circulation in the field, and the prognosis in these refractory OHCA patients has previously been shown to be dismal [9]. Extracorporeal membrane oxygenation (ECMO) support for refractory cardiac arrest patients during cardiopulmonary resuscitation (ECPR) is one of the advanced procedures used in addition to conventional standard resuscitation [10, 11]. ECPR is expected to improve clinical outcomes in out-of-hospital cardiac arrest (OHCA) patients with initial shockable rhythm (ventricular fibrillation [VF] or pulseless ventricular tachycardia [VT]) [6, 12, 13]. However, it requires intensive resources and the cost-effectiveness is uncertain.

Singapore and Osaka which is the second largest city in Japan have comparable population sizes and prehospital management; however, the frequency of ECPR differs greatly. Among the tertiary care centers in Osaka, ECPR is commonly performed in 20–30% of OHCA patients with an initial shockable rhythm [14–16]. On the other hand, in Singapore, it is rarely performed (less than 1%) because ECMO accessibility and availability are limited. Given the situation, we hypothesized that the outcomes among the OHCA patients with initial shockable rhythm but not obtaining ROSC in Singapore were different from those of Osaka. Previously, these kinds of differences have rarely been investigated. The aim of this study was to evaluate using machine learning-based prediction models, the outcomes of OHCA patients with initial shockable rhythm in Singapore compared to the expected outcomes derived from Osaka data.

## Methods

### Study design and settings

This was a secondary analysis of data from two prospective observational registries. The first was the Comprehensive Registry of Intensive Care for OHCA Survival study in Osaka (Osaka-CRITICAL), and the second was the Singapore Pan-Asian Resuscitation Outcomes Study (SG-PAROS). Details of these databases were previously published [17–20] and are described in the S-Method 1, Additional file 1. The Osaka-CRITICAL registry was a multi-institutional prospective observational study of OHCA patients whose data were obtained from 15 tertiary critical care medical centers (CCMCs) and one non-CCMC community hospital in Osaka Prefecture [21]. The data are available from 2012 to 2019. The SG-PAROS database is also a prospective, population-based registry designed to provide data on the epidemiology, management, and outcomes of OHCA in Singapore from 2010 to 2020 [19, 20]. The ethics committee of Kyoto University approved the retrospective analysis of the Osaka-CRITICAL study database (R-1045), and the Centralized Institutional Review Board and Domain Specific Review Board in Singapore granted approval for the SG-PAROS database (ref no: 2013/604/C, 2013/00929 and 2018/2937) and Domain Specific Review Board (ref no: C/10/545 and 2013/00929). Informed consent was waived due to the nature of the observational study.

This study was performed using two established databases. We first developed and validated a prediction model for the outcomes mentioned below using the data from Osaka-CRITICAL, and then, we evaluated the outcomes by comparing the actual observed outcomes in Singapore and the expected outcomes derived from Osaka using the prediction model mentioned above.

Regarding ECPR availability, tertiary critical care centers in Osaka are basically able to provide the ECPR 24/7 basis. Several papers have previously described how ECPR is currently implemented in Japan [15, 22–24], but we have included some explanation of ECPR in Additional file 1: S-Method 1. In Singapore, on the other hand, ECMO can only be implemented in two academic centers and ECPR was rarely performed during the study period.

### Study participants

This study included adult (18–74 years old) OHCA patients with initial shockable rhythm (ventricular fibrillation [VF] or pulseless ventricular tachycardia [VT]) confirmed at the scene. The inclusion criteria were similar to what is used for the selection of ECPR candidates in Osaka [12, 13]. This study excluded patients with the following: those who opted out of the study, had no prehospital record, had no resuscitation attempted, age younger than 18 years old or older than 74 years, had cardiac arrest due to external causes such as trauma, those who were not cardiac arrest at the contact with paramedics, and those whom the resuscitation was terminated in prehospital settings. In the derivation and validation of the prediction model, we included patients who met the criteria mentioned above from the Osaka-CRITICAL database between 1 July 2012 and 31 December 2019. In the main comparative analysis, we included adult OHCA patients with initial shockable rhythm as mentioned above from the SG-PAROS database between 1 July 2010 and 31 Dec 2016 and divided them into subgroups based on the cardiac rhythm at emergency department (ED) arrival: those with ROSC, those with persistently shockable rhythm, and those with nonshockable rhythm although they initially had a shockable rhythm. We excluded cases from 2017 to 2020 for the main analysis because the cardiac rhythm on ED arrival was not collected in the SG-PAROS database from 2017 to 2020, and we could not investigate the outcome by cardiac rhythm on hospital arrival for these cases. Regarding termination in Japan, the termination of resuscitation by paramedics is strictly limited. Paramedics can terminate the resuscitation only when the cardiac arrest cases are clearly considered dead such as presenting rigor mortis (the details are described in Additional file 1). Further, in the SG-PAROS database, the number of cases in which the resuscitation was terminated at the scene was only three cases during the study period (as shown in the study flowchart).

### Outcome

The primary outcome of this study was one-month survival with favorable neurological outcomes defined as Cerebral Performance Category (CPC) 1 or 2. CPC is commonly used to evaluate the neurological status after OHCA as follows (category 1, good cerebral performance; category 2, moderate cerebral disability; category 3, severe cerebral disability; category 4, coma or vegetative state; category 5, death/brain death) [25]. The secondary outcome of this study was survival admission to hospital (admission) and one-month survival. The neurological outcome and survival were evaluated by the physicians in charge or the researcher associate from clinical records, telephone, and/or face-to-face interviews [21, 26].

### Data measurement, collection, and handling of missing data

From both databases, we obtained the following clinical information: sex, age, witnessed, bystander CPR, automated external defibrillator (AED) applied by a bystander, prehospital initial cardiac rhythm, prehospital advanced airway management, adrenaline administration in the prehospital settings, cardiac rhythm on ED arrival, resuscitation time course, the disposition in the ED, application of ECMO, percutaneous coronary intervention (PCI), targeted temperature management (TTM), and outcomes. The disposition at the ED was defined as death in ED without admission, or admission to the hospital. Transfer to another hospital was categorized as admission to the hospital. The variable details are explained in Additional file 1: S-Method 2. We treated extreme outliers or contradictory data as missing. Missing data were imputed by a random forest-based imputation procedure using the “missForest” package for the study participants with eligibility [27]. We also added the detail of this imputation method and the missingness of the variables in Additional file 1: S-Method 3, S-Result 1.

## Statistical analysis

### Patient characteristics and in-hospital information

We described the patient characteristics, in-hospital information, and outcomes. Data were shown as the median and interquartile range (IQR) for continuous variables, and as numbers and percentages for categorical variables.

### Main analysis

Generally, to compare outcomes between different hospitals or regions, it is not appropriate to compare crude rates because the distribution of patient characteristics and baseline risk (case mix) is different between the populations, especially when case mix is associated with outcomes [28–30]. To deal with this, first, we developed and validated a prediction model for outcomes using the Osaka-CRITICAL data, and second, we estimated the ratio of observed to expected outcomes (OE ratio) in SG-PAROS data adjusted for patient characteristics. The OE ratio is commonly used to compare outcomes with their expected results based on the average treatment in the reference population [28, 29].

### Prediction model derivation and validation using the Osaka-CRITICAL database

In the derivation and validation of the prediction model using data from the Osaka-CRITICAL database, we divided the included patients into two cohorts: the derivation cohort from 2012 to 2017 and the validation cohort from 2018 to 2019. The derivation cohort was the reference population for comparing outcomes with Singapore. Traditionally, for risk adjustment, a logistic regression model is commonly used; however, we used a random forest (machine learning) model which has some advantages. For example, a random forest can handle nonlinear relationships in the data, reduce the risk of overfitting, and predict outcomes with high accuracy [30–33]. We derived the prediction models using random forest to incorporate the following covariates: sex, age, witnessed, bystander CPR, bystander AED, prehospital advanced airway management, prehospital adrenaline administration, cardiac rhythm on hospital arrival, and time from call to hospital. The covariates above were selected because of the availability in both datasets, clinical importance, and intention to adjust the characteristics of the case mix on hospital arrival to evaluate the potential impact of treatment strategy. Evaluating the model performance in the validation cohort, the receiver operating curves (ROCs) and the C-statistics (the value of area under the curve [AUC]) with 95% confidence interval) were calculated. Further, calibration plots using the quantile of the predicted probability were drawn to indicate the relationship between the predicted and observed probability of outcomes in the validation cohort. The other details of the derivation and validation of the models are described in the S-Method 4 in Additional file 1.

### Outcome evaluation using the SG-PAROS database

For evaluation of outcomes in Singapore, we applied the model to the SG-PAROS database to calculate the risk-adjusted predicted probability and the OE ratio with 95% confidence intervals (CI) by the subgroups based on the cardiac rhythm at ED arrival: those with ROSC, those with persistently shockable rhythm, and those with nonshockable rhythm although they initially had a shockable rhythm.

### Sensitivity analysis

To evaluate the robustness of the results, we performed the following sensitivity analysis. In sensitivity analysis 1, we also developed and validated different prediction models using the least absolute shrinkage and selection operator (LASSO) and conventional logistic regression models (Additional file 1: S-Method 5). In sensitivity analysis 2, we developed models excluding prehospital airway management and administration of drugs as predictors, considering the criticism that these variables are not appropriate as predictors as the practices regarding these procedures might differ considerably based on the region or location and they are more EMS system factors. In sensitivity analysis 3, to consider the possibility of changing results over time, we evaluated outcomes using the data from SG-PAROS 2017-2020 which were not included in the main analysis. As mentioned above, the cardiac rhythm on ED arrival was not available in the SG-PAROS 2017-2020. Thus, we developed and validated a model excluding the variable of the cardiac rhythm on ED arrival, and applied it to the SG-PAROS 2017-2020 data, and calculated the OE ratio by the prehospital ROSC (yes/no). All estimates were calculated with 95% confidence intervals (CIs). All statistical analyses were performed with R software, version 4.1.2 (R Foundation for Statistical Computing).

## Results

### Study flowchart and patient characteristics

**Fig. 1:**
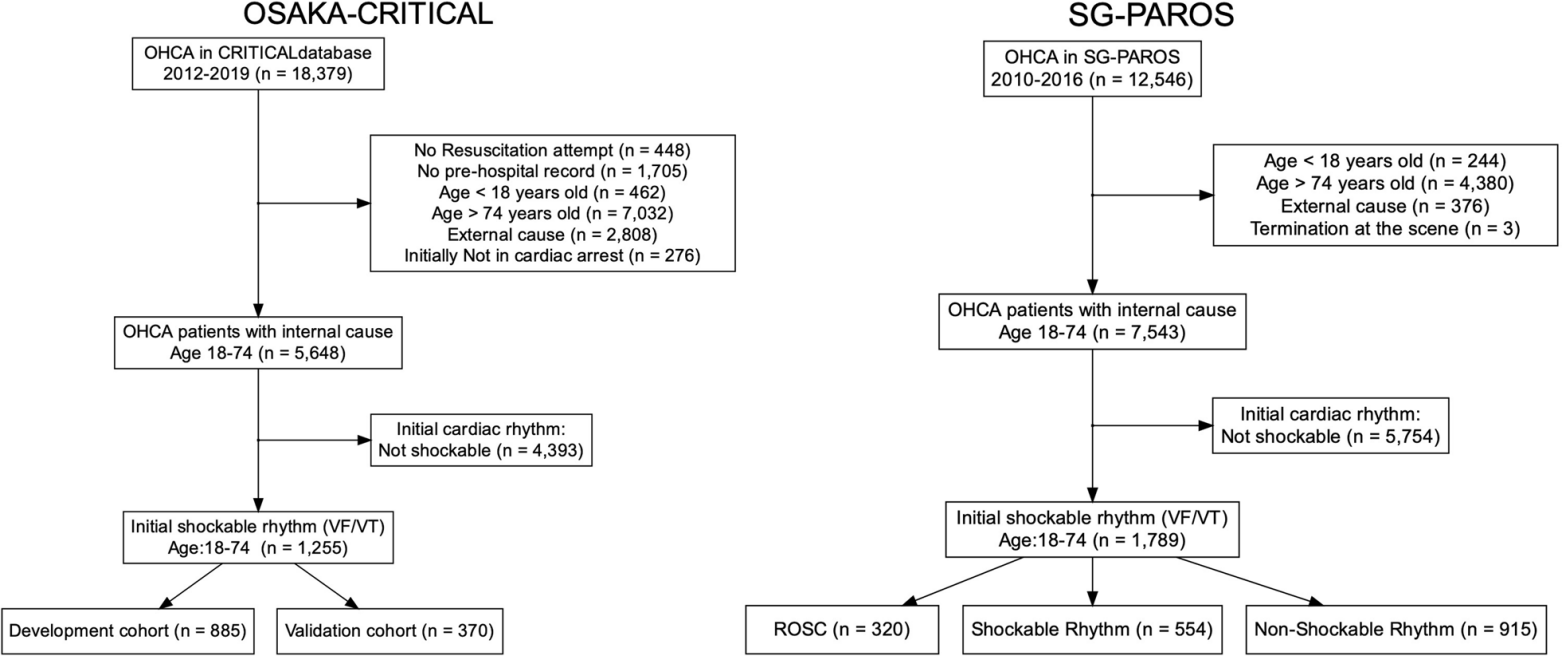
Study flowchart. OHCA, out-of-hospital cardiac arrest, VF, ventricular fibrillation, VT, ventricular tachycardia, ROSC, return of spontaneous circulation. The patients with initial shockable rhythm in the SG-PAROS database are divided by the cardiac rhythm at emergency department arrival: those with ROSC, those with persistently shockable rhythm, and those with nonshockable rhythm although they initially had a shockable rhythm

**Fig. 2:**
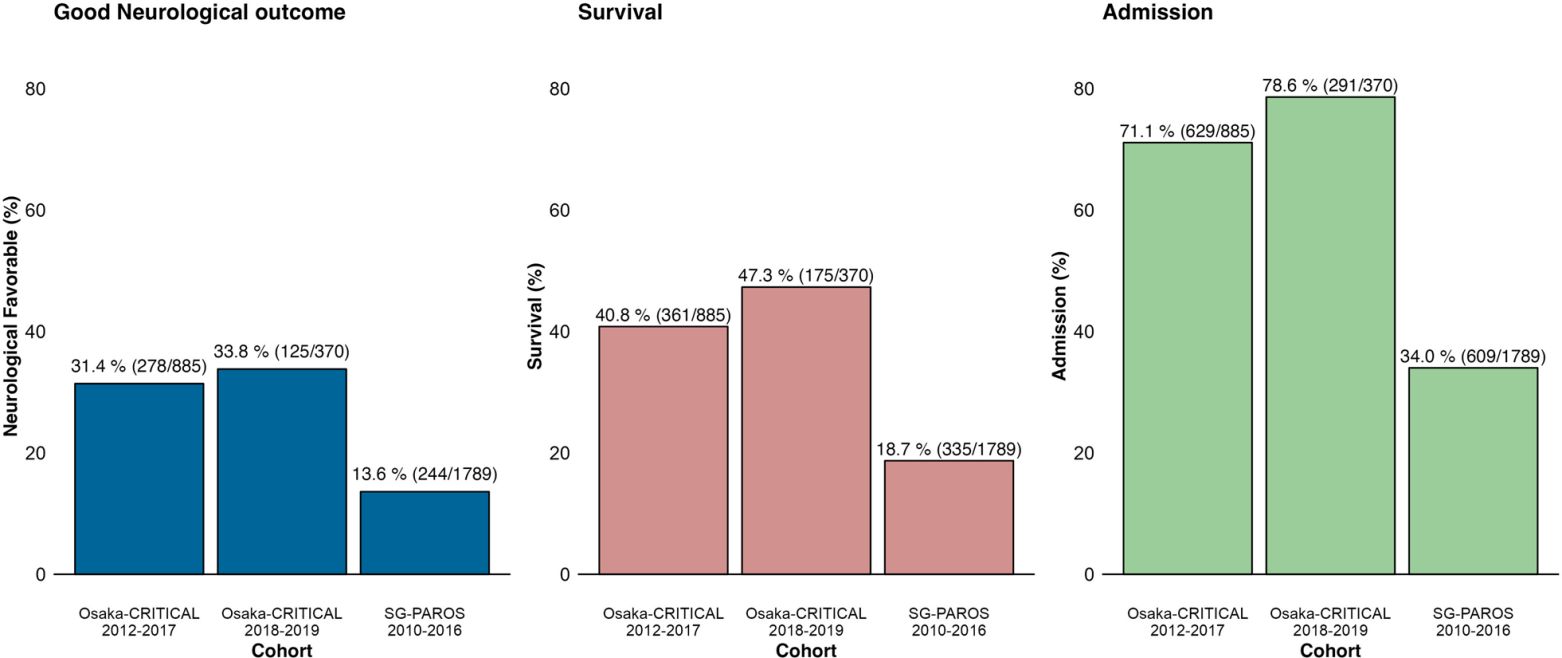
Outcomes in each cohort. Osaka-CRITICAL 2012–2017: Derivation cohort. Osaka-CRITICAL 2018–2019: Validation cohort

Of 18,379 patients registered in the Osaka-CRITICAL database, 1,255 OHCA patients with initial shockable rhythm were included in the analysis and divided into two cohorts (Derivation cohort, *n* = 885, Validation cohort, n = 370) (Fig. 1). Of 12,546 patients registered in the SG-PAROS database from 2010 to 2016, 1,789 patients with initial shockable rhythm were included in the analysis (Fig. 1). The median [IQR] age was 61 [50–68] in the derivation cohort, 60 [48–68] in the validation cohort, and 57 [50–65] in the SG-PAROS. The other basic characteristics of databases were similar except for the proportion of prehospital advanced airway management, and administration of adrenaline (Table 1). The proportion of ECMO was 35% (307/885) in the derivation cohort, 41% (150/370) validation cohort, and 0.6% (10/1,789) in the SG-PAROS cohort (Table 1). Good neurological outcomes were 31% (278/885), 34% (125/370), and 14% (244/1,789), respectively (Fig. 2). The characteristics of the cardiac rhythm on hospital arrival of the derivation cohort and the SG-PAROS were described in Table 2. Those of the validation cohort and missing values in each cohort are shown in Additional file 1: S-Results 1 and 2. Regarding PCI, TTM, and ECMO, among the patients who were the admitted to hospital, PCI was performed in 41% (257/629) of the derivation cohort, 43% (124/291) in the validation cohort, and 51% (309/609) of the SG-PAROS, TTM was applied in 58% (365/629) of the derivation cohort, 52% (150/291) in the validation cohort, and 33% (199/609) of the SG-PAROS database, and ECMO was applied in 43% (272/629) of the derivation cohort, 49% (143/291) in the validation cohort, and 1.1% (7/609) of the SG-PAROS database (Table 3). We also showed the frequencies by subgroups of cardiac rhythm on ED arrival (Table 3).

**Table 1:** Patient characteristics in the study

Characteristic	Osaka-CRITICAL 2012-2017 Derivation (*N* = 885)	Osaka-CRITICAL 2018-2019 Validation (*N* = 370)	SG-PAROS 2010-2016 (*N* = 1789)
Men	750 (85%)	327 (88%)	1546 (86%)
Age (years)	61 (50, 68)	60 (48, 68)	57 (50, 65)
Witnessed	693 (78%)	300 (81%)	1373 (77%)
Bystander CPR	419 (47%)	215 (58%)	988 (55%)
Bystander AED	46 (5.2%)	33 (8.9%)	143 (8.0%)
*Prehospital Airway*			
Intubation	218 (25%)	81 (22%)	11 (0.6%)
Supraglottic Airway	331 (37%)	120 (32%)	1557 (87%)
None	336 (38%)	169 (46%)	221 (12%)
Prehospital Drug	245 (28%)	122 (33%)	1072 (60%)
Prehospital ROSC	296 (33%)	153 (41%)	365 (20%)
Time to ED arrival (min)	30 (24, 39)	31 (26, 36)	35 (30, 40)
*Cardiac rhythm on ED arrival*			
Shockable	300 (34%)	136 (37%)	554 (31%)
Nonshockable	361 (41%)	136 (37%)	915 (51%)
ROSC	224 (25%)	98 (26%)	320 (18%)
*In-hospital procedure*			
ECMO	307 (35%)	150 (41%)	10 (0.6%)
PCI	264 (30%)	127 (34%)	309 (17%)
TTM	367 (41%)	153 (41%)	203 (11%)

Continuous variables are median and interquartile range, and categorical variables are number and percentage (%)

*CPR* Cardiopulmonary resuscitation, *AED* Automated external defibrillator, *ROSC* Return of spontaneous circulation, *ED* Emergency department, Shockable: Ventricular fibrillation and pulseless ventricular tachycardia, Nonshockable: Pulseless electrical activity and asystole, *ECMO* Extracorporeal membrane oxygenation, *PCI* Percutaneous coronary intervention, *TTM* Targeted temperature management

**Table 2:** Patient characteristics grouped by cardiac rhythm on ED arrival

	Osaka-CRITICAL 2012-2017 (Derivation cohort)			SG-PAROS 2010-2016		
Characteristic	ROSC *N* = 224	Shockable *N* = 300	Nonshockable *N* = 361	ROSC *N* = 320	Shockable *N* = 554	Nonshockable *N* = 915
Men	191 (85%)	259 (86%)	300 (83%)	271 (85%)	484 (87%)	791 (86%)
Age (years)	60 (49, 67)	61 (50, 67)	62 (51, 69)	56 (48, 64)	57 (50, 64)	58 (50, 65)
Witness	184 (82%)	237 (79%)	272 (75%)	259 (81%)	443 (80%)	671 (73%)
Bystander CPR	119 (53%)	141 (47%)	159 (44%)	192 (60%)	293 (53%)	503 (55%)
Bystander AED	15 (6.7%)	20 (6.7%)	11 (3.0%)	45 (14%)	22 (4.0%)	76 (8.3%)
*Prehospital Airway*						
Intubation	34 (15%)	86 (29%)	94 (26%)	2 (0.6%)	1 (0.2%)	8 (0.9%)
SGA	60 (27%)	92 (31%)	185 (51%)	191 (60%)	518 (94%)	848 (93%)
None	130 (58%)	122 (41%)	82 (23%)	127 (40%)	35 (6.3%)	59 (6.4%)
Prehospital Drug	38 (17%)	113 (38%)	94 (26%)	115 (36%)	409 (74%)	548 (60%)
Prehospital ROSC	213 (95%)	50 (17%)	33 (9.1%)	283 (88%)	42 (7.6%)	40 (4.4%)
Time to ED arrival (min)	29 (24, 38)	30 (23, 37)	32 (26, 40)	34 (28, 39)	34 (30, 40)	35 (30, 40)
*In-hospital procedure*						
ECMO	17 (7.6%)	179 (60%)	111 (31%)	3 (0.9%)	5 (0.9%)	2 (0.2%)
PCI	79 (35%)	114 (38%)	71 (20%)	175 (55%)	54 (9.7%)	80 (8.7%)
TTM	149 (67%)	129 (43%)	89 (25%)	104 (32%)	44 (7.9%)	55 (6.0%)
*Outcomes*						
Admission	223 (100%)	220 (73%)	186 (52%)	307 (96%)	117 (21%)	185 (20%)
Survival	200 (89%)	109 (36%)	52 (14%)	240 (75%)	57 (10%)	38 (4.2%)
Good Neurological Outcome	174 (78%)	88 (29%)	16 (4.4%)	198 (62%)	36 (6.5%)	10 (1.1%)

Continuous variables are median and interquartile range, and categorical variables are number and percentage (%)

The characteristics stratified by cardiac rhythm on ED arrival for the validation cohort are described in Additional file 1

*CPR* Cardiopulmonary resuscitation, *AED* Automated external defibrillator, *ROSC* Return of spontaneous circulation, *ED* Emergency department, Shockable: Ventricular fibrillation and pulseless ventricular tachycardia, Unshockable: Pulseless electrical activity and asystole, *ECMO* Extracorporeal membrane oxygenation, *PCI* Percutaneous coronary intervention, *TTM* Targeted temperature management

**Table 3:** Advanced procedure after admission

		Osaka-CRITICAL 2012-2017 (Derivation cohort)		
Procedures	Total *N* = 629	ROSC *N* = 223	Shockable *N* = 220	Nonshockable *N* = 186
PCI	257 (41%)	79 (35%)	109 (50%)	69 (37%)
TTM	365 (58%)	149 (67%)	127 (58%)	89 (48%)
ECMO	272 (43%)	17 (7.6%)	160 (73%)	95 (51%)

		SG-PAROS 2010-2016		
Procedures	Total *N* = 609	ROSC *N* = 307	Shockable *N* = 117	Nonshockable *N* = 185
PCI	309 (51%)	175 (57%)	54 (46%)	80 (43%)
TTM	199 (33%)	104 (34%)	41 (35%)	54 (29%)
ECMO	7 (1.1%)	3 (1.0%)	3 (2.6%)	1 (0.5%)

These tables only included the patients who were admitted to the hospital

The advanced procedures after admission for the validation cohort are described in Additional file 1: S-Results 4

*ROSC* Return of spontaneous circulation, *PCI* Percutaneous coronary intervention, *TTM* Targeted temperature management, *ECMO* extracorporeal membrane oxygenation

### Model derivation and validation

The random forest models were built using the derivation cohort from the Osaka-CRITICAL database. The details of model features are shown in Additional file 1: S-Results 4. In the validation cohort, for the good neurological outcome, the AUC of ROC of the random forest models was 0.896 [95%CI: 0.861–0.93] and the calibration plots showed good calibration performance (Additional file 1: S-Results 4). The other models' performances are also described in the (Additional file 1: S-Results 4). The OE ratios in the validation cohort implied that the observed outcomes by each cardiac rhythm on ED arrival were as expected from the derivation cohort (Additional file 1: S-Results-4).

### The observed and expected outcomes and OE ratio by cardiac rhythm on ED arrival

**Fig. 3:**
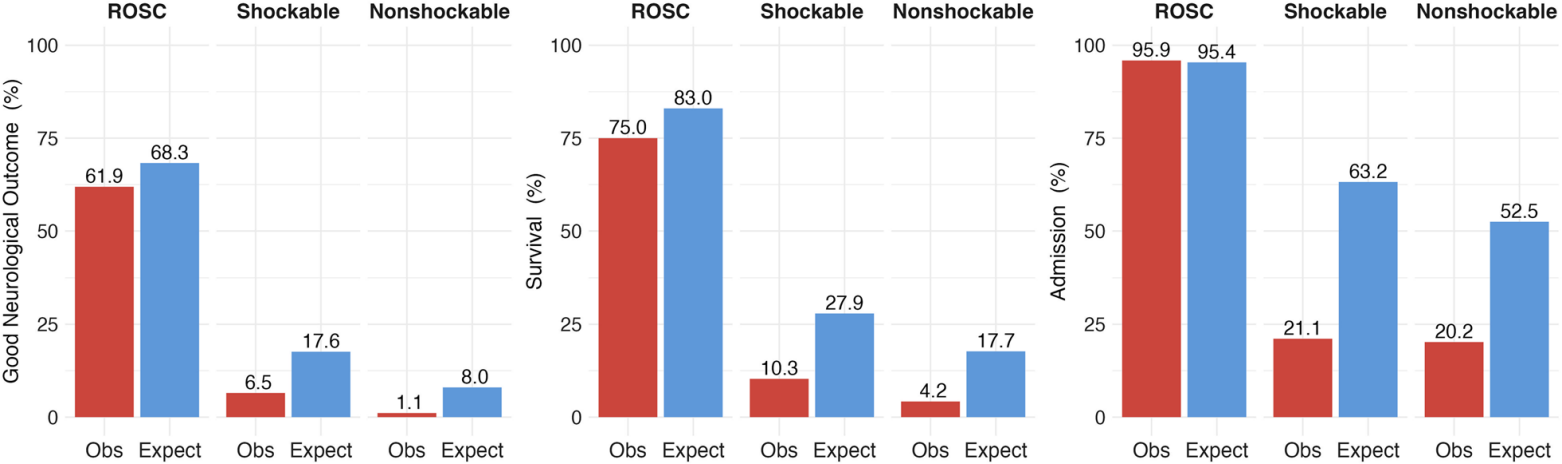
Observed and predicted outcome by cardiac rhythm on ED arrival. Obs, observed outcome, Expect, expected outcome. (Left) Good neurological outcome, (Middle) Survival outcome, (Right) Survival admission to the hospitals. For the patients with ROSC on ED arrival, the observed outcomes do not differ greatly. For the patients without ROSC, the observed outcomes are lower than expected

**Fig. 4:**
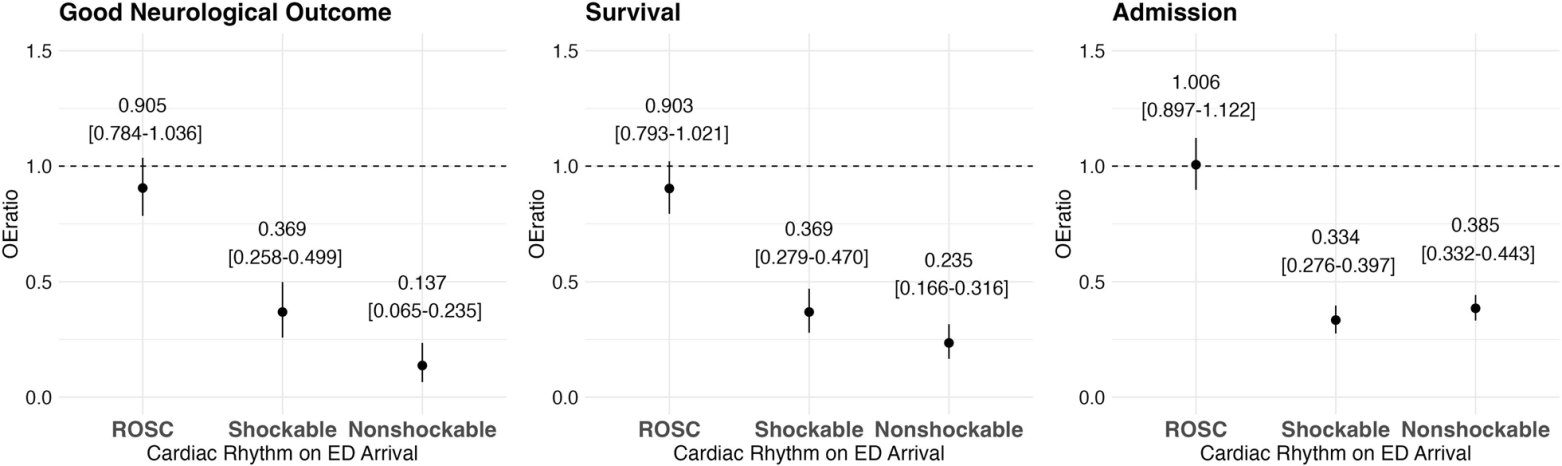
OE ratio by cardiac rhythm on ED arrival. OE ratio: Observed/expected ratio [95% confidence interval]. (Left) Good neurological outcome, (Middle) survival outcome, (Right) survival admission to the hospitals. The OE ratios for the outcomes among the patients with ROSC on ED arrival are around 1.0, implying that the outcomes in SG-PAROS are the same level as those of Osaka-CRITICAL 2012–2017. On the other hand, the OE ratios for the outcomes among the patients without ROSC on ED arrival (shockable or nonshockable) are substantially lower than 1.0, implying that the outcomes in SG-PAROS are worse than those of Osaka-CRITICAL 2012–2017

The observed and expected probabilities by ED arrival are shown in Fig. 3. Overall, observed outcomes in the patients with ROSC were similar to expected; however, those without ROSC were lower than expected. The OE ratio for good neurological outcome in SG-PAROS was 0.905 [95%CI: 0.784–1.036] in the patients with ROSC on ED arrival, 0.369 [95%CI: 0.258–0.499] in the patients with shockable rhythm without ROSC, and 0.137 [95%CI: 0.065–0.235] in the nonshockable without ROSC (Fig. 4). For survival, the OE ratio was 0.903 [95%CI: 0.793–1.021] in the patients with ROSC on ED arrival, 0.369 [95%CI: 0.279–0.470] in the patients with shockable rhythm, and 0.235 [95%CI: 0.166–0.316] in the nonshockable (Fig. 4). OE ratio for admission was 1.006 [95%CI: 0.897–1.122] in patients with ROSC on ED arrival, 0.334 [95%CI: 0.276–0.397] in the patients with shockable rhythm, and 0.385 [95%CI: 0.332–0.443] in the patients with nonshockable rhythm (Fig. 4). For these primary and secondary outcomes, the observation in the patients with ROSC on ED arrival was as expected; however, observations in the patients without ROSC were lower than expected.

### Sensitivity analysis

In sensitivity analysis 1, the logistic model and Lasso for the outcomes were also derived and validated (Additional file 1: S-Results 3–6). The results were consistent with that of the main analysis (Additional file 1: S-Results 7–9). In sensitivity analysis 2, the random forest models did not include prehospital airway management and drug administration as predictors. The OE ratios in this model were also similar to those of the main analysis (Additional file 1: S-Results 10 and 11). In sensitivity analysis 3, we evaluated the OE ratios in SG-PAROS 2017-2020. The results were consistent with the main analysis (Additional file 1: S-Results 12–14). The ECMO was also rarely performed (11/1,526, 0.7%) in SG-PAROS 2017-2020.

## Discussion

### Observations and interpretation

In this observational study, we evaluated the outcomes of OHCA patients with initial shockable rhythm in Singapore with an extremely low proportion of ECPR usage compared to those in Osaka with a high proportion of ECPR usage. The outcomes were similar between Singapore and Osaka, among the initial shockable rhythm OHCA patients with ROSC. On the other hand, the outcomes of the initial shockable rhythm OHCA patients without ROSC in Singapore were lower than expected derived from those in Osaka. This result was supported by several sensitivity analyses.

The results of this study support the idea that aggressive ECPR implementation may help to improve the outcomes of refractory OHCA patients with initial shockable rhythm. For example, the survival and neurological outcomes were almost the same between Singapore and Osaka among patients with ROSC, and these results suggest that the differences in outcomes may not be caused by differences in treatment after ROSC, such as the quality of temperature management or other intensive care. Furthermore, although hospital admission in Singapore was almost the same as expected derived from Osaka in the patients with ROSC, it was lower in patients without ROSC. This suggests that issues that make a difference in outcome may be prior to ROSC. In addition, the characteristics and important factors available at ED arrival were included in the model for risk adjustment; therefore, outcome differences were likely due to factors after hospital arrival. We did observe in this study that post-resuscitation care such as PCI and TTM were different between them; however, the overall frequency of these post-resuscitation treatments were not hugely different among all the OHCA patients who were admitted to hospitals in both datasets (Table 3). On the other hand, ECMO was rarely performed in Singapore but it was aggressively implemented in Osaka. Although other differences in post-resuscitation care such as how fast PCI or TTM was initiated, or the implementation of post-resuscitation care might have influenced the results, it may be reasonable to consider that an aggressive ECPR implementation policy contributed to improving the outcomes of refractory OHCA patients with initial shockable rhythm. Furthermore, some previous studies also suggested results consistent with this study. A nationwide OHCA registry in Japan reported that in patients with initial shockable rhythm who continued to have a shockable rhythm on hospital arrival (age: median [IQR], 66 [54–75] years), good neurological outcome and survival were 17.4% and 26.6%, respectively, and the proportion of ECMO implementation for them was 54.0% [34]. These results were almost the same in the Osaka-CRITICAL database.

One of the strengths of this study is that it was like a “natural experiment,” and the results are worth considering. Previously, one observational study attempted to investigate the outcome of OHCA patients between facilities with and without ECPR capability in the same area [35]. However, these studies could not eliminate the risk of selection bias based on different indications for ECPR in the areas. On the other hand, this study investigated the outcomes in Singapore and Osaka, which are large urban cities in Asia, and the patients in this study had similar characteristics such as age, the proportion of bystander CPR, and implementation of AED, and the percentage of PCI and TTM performed among the admitted patients were not so different; however, the proportion of ECPR implementation was very different. Although there might be some unmeasured confounding factors, this was like an ideal “natural experiment.”

One other strength was that this study investigated the outcome differences at the systems level. Previously, one RCT (the ARREST trial) showed a beneficial effect of ECPR, but the latest RCT did not [36, 37]. However, critics of this latest RCT suggest that the healthcare system was not adequately set up to provide high-quality ECPR, as the time taken to start ECPR was rather long (the median [IQR] duration from the arrest to the start of ECMO was 74 [63, 87] minutes) [37]. On the other hand, in the ARREST trial, the mean (standard deviation) time to start ECMO was 59 (28) minutes [36]. The outcomes of OHCA patients treated with ECPR are time sensitive, and a longer duration between the arrest and the start of ECPR has worse outcomes [38]. Successful ECPR essentially requires a chain of appropriate actions including immediate high-quality bystander CPR and AED, early recognition of the indications for ECPR, prompt transfer to a high-level facility with experienced staff, and well-trained teamwork for cannulation of ECMO, resulting in a shorter time to initiation of ECPR. Accordingly, an ECPR strategy should be evaluated at the system level. In the Osaka-CRITICAL database, the median [IQR] duration from ambulance call to start ECPR was 53 [45, 64] minutes [15], and the quality of systems providing ECPR in Osaka was relatively high. In this regard, although this study did not directly evaluate the association between the ECPR and outcomes, it may provide evidence that a good quality system of early ECPR implementation may lead to favorable outcomes in refractory OHCA patients with initial shockable rhythm.

### Clinical implications

Although the results of this study suggest the potential of ECPR to improve outcomes, we believe that there should still be caution before adopting an aggressive ECPR policy in all situations because of several concerns. One of the concerns is that the population which is suitable for ECPR is still unclear. The indications for ECPR in the Osaka-CRITICAL study were decided by each hospital protocol or physician in charge, but there were no strictly defined inclusion criteria. Previously, some studies were investigating which population is suitable for ECPR [15, 16, 39–41], without a clear consensus.

The other important concern is that it is challenging to establish a well-functioning, sustainable system. As mentioned, successful ECPR requires a well-designed healthcare and education system [42–44]. However, building such a system may require time, cost, education, human resources, a skilled team, and the accumulation of experience [42–44]. Furthermore, since ECPR is a time-critical treatment [38], it is challenging to build a system providing ECPR equally to people living in different parts of a country, where the time and distance to hospitals vary [45]. Also, the implementation of ECMO itself requires a lot of costs [46, 47], which can be a burden for patients and health insurance systems. Previously, some studies of the cost-effectiveness of ECPR compared with conventional CPR indicated that the ECPR was acceptable based on the cost-effectiveness in Japan and some other countries [46, 47]. However, these studies only considered the cost of the ECPR itself, not how much funding is necessary to build the system and sustain it. In Japan, medical cost is a huge burden and there are some concerns about how to sustain the current healthcare system [48]. Furthermore, there are other priorities for improving the outcomes of the patients with OHCA such as promoting basic life support education or improving the accessibility of AED in public places [49]. Accordingly, further research is warranted to evaluate the cost-effectiveness of any strategy.

### Limitations

There were several limitations. First, we used the Osaka-CRITICAL database as a reference, which was not a population-based registry, as the patients are from tertiary care hospitals, whereas the SG-PAROS was a population-based registry. Although 99% of the patients in both databases were patients transferred to tertiary care hospitals, there might be the risk of selection bias in the reference population. Second, there might be the risk of bias due to unmeasured factors prior to hospital arrival that were not included in the model such as past medical history, frailty, socioeconomic status, or differences in prehospital emergency systems between Singapore and Osaka. We could not account for the clinical impact of these factors. However, we suggest that if unmeasured factors influenced the result, the magnitude was not so huge because the outcomes of patients obtaining ROSC in Singapore were almost the same as in Osaka. If the prehospital emergency systems in Singapore had a huge negative impact on the outcomes, the outcomes of patients with ROSC would also have been worsened. Third, there was some risk of overfitting of the prediction model. However, we performed some sensitivity analysis, and it supported that the main results were robust, even if the model was changed slightly or the statistical procedure was changed. Fourth, this study could not investigate the direct association between ECPR policy and outcome. This study only suggested that something was different between Osaka and Singapore after hospital arrival which led to worse outcomes among the patients with OHCA who had initial shockable rhythm but did not achieve ROSC. For example, some other resuscitation procedures in the ED, the timing of the decision to terminate resuscitation, or other differences in post-resuscitation care such as how fast PCI or TTM was initiated, or the implementation of post-resuscitation care bundle might cause differences. Unfortunately, we could not compare the details of resuscitation due to lack of data granularity. Further studies are necessary to confirm the generalizability of these results.

## Conclusion

This observational study found that the outcomes for patients with initial shockable rhythm but who did not obtain ROSC on hospital arrival in Singapore were lower than expected from Osaka. We hypothesize this is mainly due to differences in the use of ECPR.

### Supplementary Information

**Additional file 1.** Supplementary Methods and Results.

## Data Availability

This study's datasets and/or analyses are not publicly available because the ethics committee did not permit it.
