# Supplementary material for: Outcome assessment for out-of-hospital cardiac arrest patients in Singapore and Japan with initial shockable rhythm
Source: Crit Care. 2023 Sep 12;27:351. doi: 10.1186/s13054-023-04636-x (PMC10496207; doi:10.1186/s13054-023-04636-x)
Supplement: Supplementary file 1 — Additional file 1. Supplementary Methods and Results. [file 13054_2023_4636_MOESM1_ESM.docx]

**Additional file 1**

**Title:** Outcome Assessment for Out-of-Hospital Cardiac Arrest Patients in Singapore and Japan with Initial Shockable Rhythm

**Contents**

| **S-Method 1** | **Description of databases and how to perform ECPR in Japan** |
| --- | --- |
| **S-Method 2** | **Description of the variables in the database** |
| **S-Method 3.** | **Missing imputation** |
| **S-Method 4.** | **Model derivation and validation** |
| **S-Method 5.** | **Sensitivity analysis** |
| **S-Results 1.** | **Missing of the data** |
| **S-Results 2.** | **Patients characteristics by cardiac rhythm on ED arrival in the validation data** |
| **S-Results 3.** | **Model features** |
| **S-Results 4.** | **The advanced procedures after admission in validation cohort** |
| **S-Results 5.** | **Model performance in the validation cohort** |
| **S-Results 6.** | **The OE ratio by the cardiac rhythm on ED arrival in the validation cohort** |
| **S-Results 7.** | **Relationship observation and prediction in the SG-PAROS** |
| **S-Results 8.** | **Observation and Prediction by cardiac rhythm on ED arrival in SG-PAROS data** |
| **S-Results 9.** | **OE ratios by cardiac rhythm on ED arrival in SG-PAROS data** |
| **S-Results 10.** | **The results of sensitivity analysis (The model in Sensitivity analysis 2)** |
| **S-Results 11** | **The results of sensitivity analysis (Outcomes in Sensitivity analysis 2)** |
| **S-Results 12.** | **The results of sensitivity analysis (Characteristics in Sensitivity analysis 3)** |
| **S-Results 13.** | **The results of sensitivity analysis (The model in Sensitivity analysis 3)** |
| **S-Results 14.** | **The results of sensitivity analysis (Outcomes in Sensitivity analysis 3)** |

**S-Method 1. Description of databases and how to perform ECPR in Japan**

1. **Osaka-CRITICAL database**

The Comprehensive Registry of Intensive Care for OHCA Survival study in Osaka (Osaka-CRITICAL) database is a multi-institutional prospective observational study of out-of-hospital cardiac arrest (OHCA) patients in Osaka Prefecture, Japan. Pre-hospital data were obtained from the All-Japan Utstein Registry of the Fire and Disaster Management Agency (FDMA).^1-4^ In-hospital data were obtained from 15 tertiary critical care medical centres (CCMCs) and one non-CCMC community hospital with an emergency department, all located in Osaka prefecture in Japan. Osaka Prefecture is an urban region, with an area of 1,905 km^2^, and it has a residential population of approximately 8.8 million in 2015.^5^ In Osaka Prefecture, 7,500 OHCA cases occur every year,^6^ and approximately one in four OHCA patients (approximately 2,000 cases or more) have been registered every year from 2012 to 2019. This registry is ongoing, with an undefined study period. In-hospital data were recorded by the physicians in charge of the patients and registered by the physicians or medical administrators using a predefined online form. Finally, the working group checked and confirmed the quality of the data. If the data were incomplete, then they were returned to each institution, and the data were then completed. ^4^ A detailed description of the All-Japan Utstein Registry of FDMA and the CRITICAL study has been previously published. ^4^

Regarding termination in Japan, the termination of resuscitation by paramedics is strictly limited. Paramedics can terminate the resuscitation only when the cardiac arrest cases are apparently considered dead, such as the neck or trunk has been amputated, the whole body is decomposing or met the following ALL six criteria: GCS1-1-1, respiratory arrest, cardiac arrest with asystole, pupil is fix and dilated, body temperature is lower than the normal range, and rigor mortis is observed. In such a case, paramedics can consult with the medical director and terminate the resuscitation. Otherwise, paramedics must transfer all the OHCA patients to the hospital. During the study period, the do-not-attempt-resuscitation order was generally not applicable to resuscitations performed by paramedics. Accordingly, we guess that there may be no case that the resuscitation was terminated at the scene and that was not transferred to the hospital among the OHCA patients with initial VF because they don’t match the requirement of cardiac arrest with asystole.

1. **SG-PAROS database**

Singapore is a small island city-state located in Southeast Asia, just north of the equator. It is known for its high population density, modern infrastructure, and diverse population. The population of Singapore was approximately 5.7 million people. The total land area of Singapore is approximately 719.1 square kilometers.

The Pan-Asian Resuscitation Outcomes Study (PAROS) is a registry that collects data on out-of-hospital cardiac arrests (OHCAs) that occur in the Asia-Pacific region. It was established in 2009 and includes all OHCAs that are either treated by emergency medical services (EMS) or presented to emergency departments (EDs). The data collected by PAROS follows the Utstein-style guidelines, which provide a standardized way to report data on OHCAs. This includes information on the prehospital care and medical procedures that were administered, as well as the outcomes of the OHCAs. The purpose of PAROS is to improve the understanding of OHCAs in the Asia-Pacific region and to help identify ways to improve the outcomes of these events. ^7^ In this database, 99% of the cardiac arrest patients were transferred to the 8 tertiary care hospitals in Singapore.

1. **How to perform ECPR in Japan**

In Japan, the first large prospective observational study (SAVE-J study) indicated the potential favorable effect of ECPR by assessing 46 institutions during 2008-2011. ^8,9^ Since then, eligibility criteria reported in the SAVE-J study (initial shockable rhythm, time from call to hospital arrival is within45 minutes, and age younger than 75 years old) have been conventionally considered one of the important references in Japan. ^8,9^ Although some of the guidance exists, the defined formal criteria are lacking in almost half of the tertiary care hospitals, and actual indication is decided by a physician in charge of the patients in these hospitals. ^10,11^ The implementation of ECPR is usually just after arrival at the tertiary care hospital. In most cases, ECMO priming starts before the patients' arrival or within 5 minutes after arrival.^11^ Generally, a stand-by call of ECPR candidate from the ambulance assembles the staff including the emergency physician, cardiologists, perfusionist, and nurses to the emergency department, and prepare the emergency department or catheter suite. About half of the hospitals implement the ECPR in the catheter suite, while the others do it in the emergency department or emergency department with angio facilities. ^11^ Generally, vascular access is obtained using percutaneous access with ultrasonography. ^11^ In most cases, emergency physicians and/or cardiologists perform the cannulation. ^11^ During the procedures, most hospitals limit the defibrillation for persistent shockable rhythm, if it is applied in the prehospital settings, and sometimes drug administration is also omitted. Generally, the time from the patient's arrival at the hospital to run the ECMO is around 15-30 minutes (the median 22 minutes interquartile range [15-32] minutes). ^10^ The prehospital ECPR is experimentally provided by some hospitals using physician-staffed ambulances; however, actual cases are very limited.

**S-Method 2. Description of the variables in the database**

| Variable | Description |
| --- | --- |
| Basic demographics | |
| Sex | Sex (Men/Women) |
| Age | Age (years) |
| Prehospital information (Utstein style) | |
| Witnessed | Witness of collapse (yes/no) |
| Bystander CPR | Bystander CPR (yes/ no or not registered) |
| Initial cardiac rhythm | The cardiac rhythm initially confirmed by paramedics at the scene (VF or VT/PEA/Asystole) |
| Bystander AED | Defibrillation performed by Bystander (yes/ no or not registered) |
| Prehospital advanced airway management | Advanced airway management performed by paramedics (Intubation/Supraglottic airway/None) |
| IV adrenaline | Administrations of adrenaline via iv route by paramedics (yes/ no or not registered) |
| Prehospital ROSC | ROSC at the scene or during the transportation (yes/ no or not registered) |
| Time from Call to Hospital | The time from emergency call to the patients arrived at hospital (minute) |
| In-hospital information | |
| Initial Cardiac Rhythm ED on Arrival | Initial cardiac rhythm confirmed on ED arrival (ROSC/Shockable/Nonshockable) |
| Disposition in the ED | Death in ED without admission, or admission to the hospital. The transfer to another hospital was categorized as admission to the hospital. |
| ECMO | ECMO was performed in ED yes/no or not registered. |
| PCI | The emergency PCI was performed yes/no or not registered |
| TTM | TTM was performed yes/no or not registered. |
| Survival | The status at the 30th-day post-arrest or the time of discharge. Survival: The patient was discharged alive or remains in the hospital on the 30th day post-arrest. Dead: Died in hospital. |
| Neurological outcome | The status at the 30th-day post-arrest or the time of discharge. Good: Survival with favorable neurological outcomes defined as CPC 1 or 2, Poor: the status CPC3or 4, or death (CPC5) |

CPR, Cardiopulmonary resuscitation, VF, Ventricular fibrillation, VT, Ventricular tachycardia, PEA: Pulseless electrical activity, ROSC, Return of spontaneous circulation, AED, Automated external defibrillator, ECMO, Extracorporeal membrane oxygenation, PCI, Percutaneous coronary intervention, TTM, Targeted temperature management. CPC, Cerebral Performance Category.

**S-Method 3. Missing imputation**

To address missing variables, we utilized the "missForest" package, a machine learning-based imputation technique, to impute the missing values. ^12,13^ This imputation technique is a nonparametric algorithm that can accommodate nonlinearities and interactions, and the single point estimates can be generated accurately by a random forest model. ^12,13^ The use of a random forest model has the advantage of being able to handle both continuous and categorical responses, requiring minimal tuning, and providing an internally cross-validated error estimate. This imputation technique has been shown to be reliable and valid compared to other imputation methods such as k-nearest neighbors’ imputation or multivariate imputation using chained equations. ^12,13^ Missingness was imputed using all predictors and outcomes.

**S-Method 4. Model derivation and validation**

**Derivation and validation cohort**

We divided the included patients in Osaka-CRITICAL into two groups: a derivation cohort from 2012-2017 and a validation cohort from 2018-2019. The purpose of this division was to validate the prediction model by confirming its generalizability to a slightly different patient population. Generally, external validation of the prediction model requires a different patient spectrum. ^14,15^ We chose to divide the data chronologically because they wanted to predict the outcome if OHCA patients in other areas were transferred to hospitals in the Osaka-CRITICAL database and received the average treatment as a reference, and by separating the data in this way, the calendar year in the derivation cohort was closer to the targeted included patients in the SG-PAROS study. Regarding the sample size, previous literature suggested at least 100 events for validating prediction models; thus, the validation cohort was set based on the number of cases with favorable neurological outcomes, and the rest of the available data was used for the development cohort.^16^

**Model derivation**

Based on previous studies, we chose the random forest model in the main analysis which is one of the most common models. ^17-20^ Random forest is an ensemble learning method that consists of hundreds or thousands of decision trees.^21^ It trains each one on a slightly different set of observations using bootstrapping, and the final predictions are made by averaging the predictions of each individual tree. For developing the random forest, we performed optimization of the hyperparameters by grid search strategy using the “ranger” and “caret” packages. ^22,23^ To understand the contribution of predictors to the models, we showed that the variable importance scaled as the maximum value is 100.^23,24^

**Model evaluation in the validation cohort**

For the assessment of predictive performance, developed models were applied to the validation cohort as external validation. The receiver operating curves (ROCs) were drawn, and the area under the curve (AUC) with the 95% confidence interval (95% CI) were calculated as discrimination. The model’s performance was also evaluated based on the C index, the Nagelkerke R^2^ value, calibration intercept and slope, and the Brier score.^14^ Calibration plots were also created to graphically indicate the association between the predicted and observed outcome using tertile.

**S-Method 5. Sensitivity analysis**

**Sensitivity analysis 1. Different models using the logistic model and Lasso**

Based on previous studies, ^17,19,20,25-29^ we chose the Lasso and conventional logistic regression model to develop the prediction model in the derivation cohort as a sensitivity analysis. Lasso regularization can choose a few relevant variables and ignore others to reduce the model complexity and prevent overfitting.^30-32^ This features selection can also enable us to interpret the model. For the training, we used 10-fold cross-validation by the “glmnet” package ^33^ to select the optimal value of the penalty parameter (lambda) and calculated the beta coefficient of the selected variables. The variables and outcomes were the same as the main analysis. The assessment of predictive performance was performed also in the same manner as the main analysis.

**Sensitivity analysis 2. Different models without airway management and drug administration**

We understand some criticism that prehospital airway management and prehospital drug administration should not be included in the prediction models because they are kinds of intervention and not predictors. Accordingly, we developed the random forest model similar to the main analysis but excluding the variable of prehospital airway management and administration of the drug as predictors.

**Sensitivity analysis 3. Different period**

In sensitivity analysis 3, to consider the possibility of changing the results over time, we evaluated the outcomes using the data from SG-PAROS 2018-2020 which were not included in the main analysis. As mentioned in the method part, the cardiac rhythm on ED arrival is not available in the SG-PAROS 2017-2020. Thus, we developed and validated the model excluding the variable of the cardiac rhythm on ED arrival as same as in the main analysis, and it applied to the SG-PAROS 2017-2020, and we calculated the OE ratio by the group of prehospital ROSC (yes/no).

**S-Results 1. Missing of the data**

| Characteristic | Osaka-CRITICAL  2012-2019  (N = 1,255) | SG-PAROS  2010-2016  (N = 1,789) |
| --- | --- | --- |
| Men | 0 (0%) | 0 (0%) |
| Age | 0 (0%) | 0 (0%) |
| Witnessed | 0 (0%) | 0 (0%) |
| Bystander CPR | 0 (0%) | 0 (0%) |
| Bystander AED | 0 (0%) | 0 (0%) |
| Prehospital Airway Type | 256 (20%) | 0 (0%) |
| Prehospital Drug | 0 (0%) | 0 (0%) |
| Prehospital ROSC | 0 (0%) | 0 (0%) |
| Time to ED arrival | 23 (1.8%) | 13(0.7%) |
| Cardiac rhythm on ED arrival | 0 (0%) | 0 (0%) |
| Outcome |  |  |
| Disposition of ED | 0 (0%) | 0 (0%) |
| 30-day Survival | 0 (0%) | 0 (0%) |
| 30-day Neurological outcome | 0 (0%) | 0 (0%) |

Number and percentage (%). CPR, Cardiopulmonary resuscitation, Shockable: Ventricular fibrillation and pulseless ventricular tachycardia, Unshockable: Pulseless electrical activity and asystole, ROSC, Return of spontaneous circulation, AED, Automated external defibrillator, ROSC, Return of spontaneous circulation, ED: Emergency department.

**S- Results 2. Patients characteristics by cardiac rhythm on ED arrival in the validation data**

|  | Osaka-CRITICAL 2018-2019  (Validation cohort) | | |
| --- | --- | --- | --- |
| Characteristic | ROSC  N = 98 | Shockable  N = 136 | Nonshockable  N = 136 |
| Men | 85 (87%) | 126 (93%) | 116 (85%) |
| Age (years) | 60 (47, 67) | 62 (50, 69) | 56 (48, 67) |
| Witness | 87 (89%) | 106 (78%) | 107 (79%) |
| Bystander CPR | 61 (62%) | 78 (57%) | 76 (56%) |
| Bystander AED | 12 (12%) | 14 (10%) | 7 (5.1%) |
| Pre-hospital Airway |  |  |  |
| Intubation | 10 (10%) | 37 (27%) | 34 (25%) |
| SGA | 22 (22%) | 43 (32%) | 56 (41%) |
| None | 66 (67%) | 56 (41%) | 46 (34%) |
| Pre-hospital Drug | 17 (17%) | 51 (38%) | 54 (40%) |
| Pre-hospital ROSC | 96 (98%) | 37 (27%) | 20 (15%) |
| Time to ED arrival (min) | 29 (24, 36) | 30 (25, 34) | 32 (27, 37) |
| In-hospital procedure |  |  |  |
| PCI | 37 (38%) | 55 (40%) | 35 (26%) |
| TTM | 60 (61%) | 57 (42%) | 36 (26%) |
| ECMO | 8 (8.2%) | 87 (64%) | 55 (40%) |
| Outcome |  |  |  |
| Admission | 96 (98%) | 112 (82%) | 83 (61%) |
| Survival | 90 (92%) | 57 (42%) | 28 (21%) |
| Good Neurological Outcome | 77 (79%) | 36 (26%) | 12 (8.8%) |

Continuous variables are median and interquartile range, and categorical variables are number and percentage (%).

CPR, Cardiopulmonary resuscitation, AED, Automated external defibrillator, ROSC, Return of spontaneous circulation, ED, Emergency department, Shockable: Ventricular fibrillation and pulseless ventricular tachycardia, Unshockable: Pulseless electrical activity and asystole, ECMO, Extracorporeal membrane oxygenation, PCI, Percutaneous coronary intervention, TTM, Targeted temperature management

**S-Results 3. The advanced procedures after admission in validation cohort**

|  |  | Osaka-CRITICAL 2018-2019  (Validation cohort) | | |
| --- | --- | --- | --- | --- |
| Procedures | Total  N = 291 | ROSC  N = 96 | Shockable  N = 112 | Nonshockable  N = 83 |
| PCI | 124 (43%) | 36 (38%) | 53 (47%) | 35 (42%) |
| TTM | 150 (52%) | 59 (61%) | 55 (49%) | 36 (43%) |
| ECMO | 143 (49%) | 8 (8.3%) | 81 (72%) | 54 (65%) |

These tables only included the patients who were admitted to the hospital.

ROSC, Return of spontaneous circulation, PCI, Percutaneous coronary intervention, TTM, Targeted temperature management.

**S-Results 4. Model features**

**Random Forest (Variable importance)**

| **Predictor** | **Neurological** | **Survival** | **Admission** |
| --- | --- | --- | --- |
| Sex (Women) | 2.3 | 1.1 | 0 |
| Age | 6.7 | 3.9 | 4.3 |
| Witness (Yes) | 1.9 | 1.7 | 5.6 |
| Bystander CPR (Yes) | 0.8 | 1.2 | 1.7 |
| Bystander AED (Yes) | 0 | 0 | 2.1 |
| Pre-hospital airway (None) | 21.9 | 12.7 | 22.9 |
| Pre-hospital airway (Supraglottic airway) | 6.5 | 6.7 | 6.3 |
| Pre-hospital drug administration (Yes) | 15.8 | 9.6 | 9.5 |
| Prehospital ROSC (Yes) | 96.7 | 100 | 100 |
| Time to ED arrival | 4 | 8.4 | 9.4 |
| Cardiac rhythm on ED arrival (ROSC) | 100 | 79.9 | 70.4 |
| Cardiac rhythm on ED arrival (Shockable) | 18 | 11.4 | 26.2 |

Variable importance scaled as the maximum value is 100.

**Logistic model (beta-coefficient)**

| **Predictor** | **Neurological** | **Survival** | **Admission** |
| --- | --- | --- | --- |
| Intercept | -1.196 | -0.509 | 0.412 |
| Sex (Women) | 0.071 | 0.127 | 0.408 |
| Age | -0.045 | -0.023 | -0.009 |
| Witness (Yes) | -0.362 | -0.032 | 0.265 |
| Bystander CPR (Yes) | 0.114 | -0.143 | 0.166 |
| Bystander AED (Yes) | 0.134 | 0.085 | 0.098 |
| Prehospital airway (None) | 1.233 | 0.718 | 0.352 |
| Prehospital airway (Supraglottic airway) | 1.187 | 0.896 | 0.073 |
| Prehospital drug administration (Yes) | -0.851 | -0.504 | -0.132 |
| Prehospital ROSC (Yes) | 2.049 | 2.047 | 2.595 |
| Time to ED arrival | -0.012 | -0.021 | -0.011 |
| Cardiac rhythm on ED arrival (ROSC) | 3.046 | 2.504 | 3.351 |
| Cardiac rhythm on ED arrival (Shockable) | 2.371 | 1.256 | 0.846 |

CPR, Cardiopulmonary resuscitation, Shockable: Ventricular fibrillation and pulseless ventricular tachycardia, Unshockable: Pulseless electrical activity and asystole, ROSC, Return of spontaneous circulation, AED, Automated external defibrillator, ROSC, Return of spontaneous circulation, ED: Emergency department.

**Lasso (beta-coefficient)**

| **Predictor** | **Neurological** | **Survival** | **Admission** |
| --- | --- | --- | --- |
| Intercept | -3.914 | -3.604 | -3.558 |
| Sex | 0 | 0 | 0.272 |
| Age | -0.04 | -0.019 | -0.006 |
| Witnessed | -0.196 | 0 | 0.184 |
| Bystander CPR | 0.092 | -0.003 | 0.095 |
| Bystander AED | 0.084 | 0 | 0 |
| Pre-hospital Airway Type | 0.315 | 0.266 | 0 |
| Pre-hospital Drug | -1.166 | -0.634 | -0.146 |
| Pre-hospital ROSC | 3.173 | 2.991 | 3.313 |
| Time to ED arrival | -0.013 | -0.018 | -0.012 |
| Cardiac rhythm on ED arrival | 1.039 | 0.57 | 0.424 |

CPR, Cardiopulmonary resuscitation, Shockable: Ventricular fibrillation and pulseless ventricular tachycardia, Unshockable: Pulseless electrical activity and asystole, ROSC, Return of spontaneous circulation, AED, Automated external defibrillator, ROSC, Return of spontaneous circulation, ED: Emergency department.

**S-Results 5. Model performance in the validation cohort**

| **Model** | **Outcome** | **C-stat** | **R2** | **Brier** | **Intercept** | **Slope** |
| --- | --- | --- | --- | --- | --- | --- |
| Random Forest | Neurological | 0.896 | 0.548 | 0.12 | -0.025 | 1.286 |
| Random Forest | Survival | 0.849 | 0.467 | 0.156 | 0.221 | 1.124 |
| Random Forest | Admission | 0.778 | 0.230 | 0.145 | 0.330 | 0.988 |
| Logistic regression | Neurological | 0.897 | 0.558 | 0.121 | -0.185 | 0.915 |
| Logistic regression | Survival | 0.849 | 0.457 | 0.157 | 0.058 | 0.835 |
| Logistic regression | Admission | 0.796 | 0.220 | 0.142 | 0.444 | 0.635 |
| Lasso | Neurological | 0.879 | 0.505 | 0.131 | -0.238 | 0.925 |
| Lasso | Survival | 0.824 | 0.400 | 0.166 | 0.011 | 0.831 |
| Lasso | Admission | 0.788 | 0.216 | 0.142 | 0.401 | 0.740 |

C-stat: C-statistics

**ROC (Receiver operating curve) in the main analysis and in the sensitivity analysis 1**

**Calibration plot in the main analysis and in the sensitivity analysis 1**

**
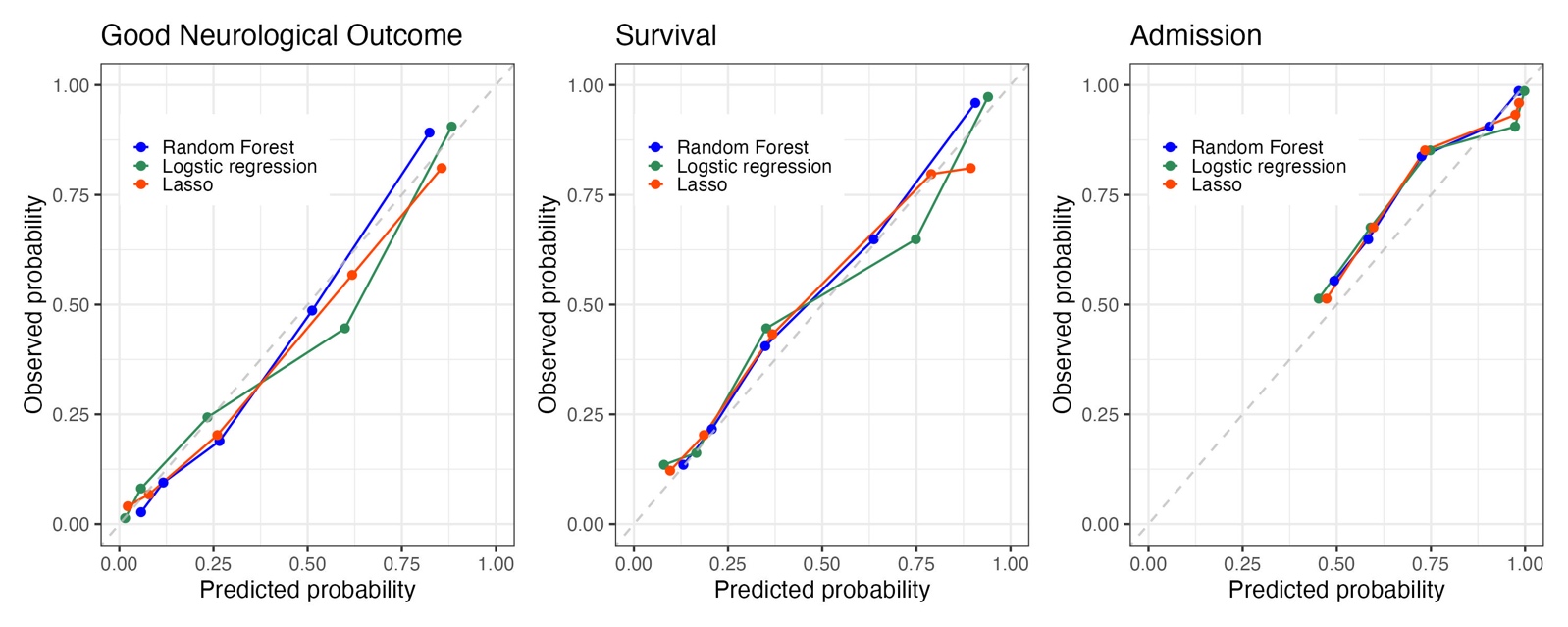
**

Diagonal lines imply the perfect agreement between prediction and observation.

All the calibration plots are almost along with the diagonal lines.

**S-Results 6. The OE ratio by the cardiac rhythm on ED arrival in the validation cohort**

**Random forest**


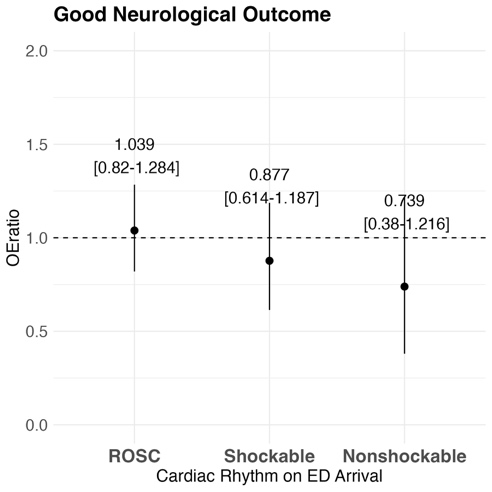

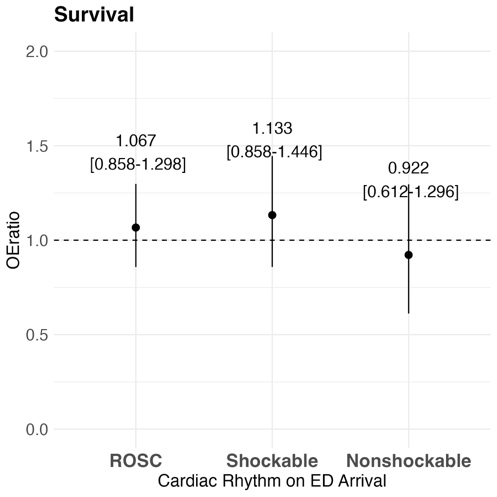

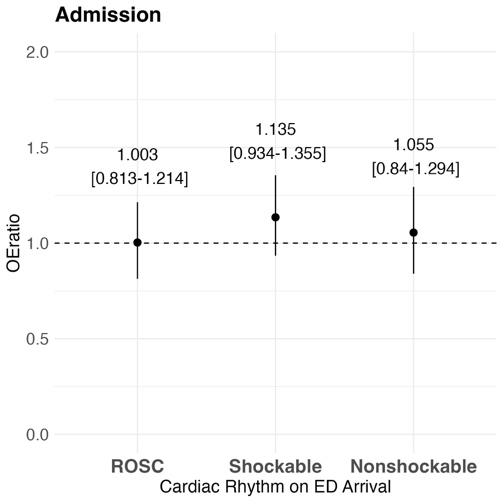


**Logistic model**


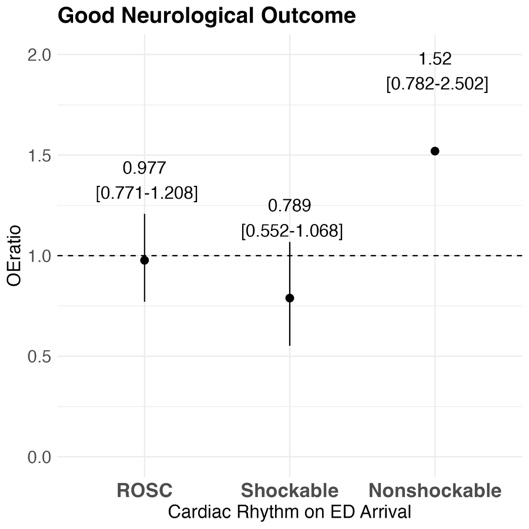

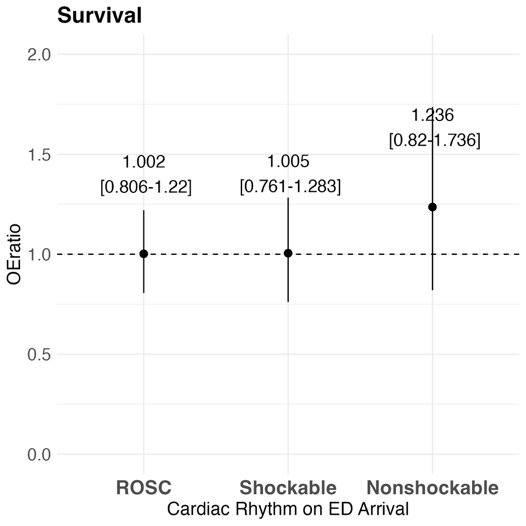

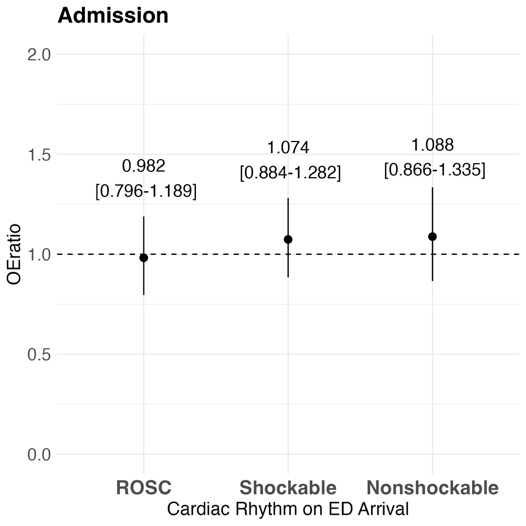


**Lasso**


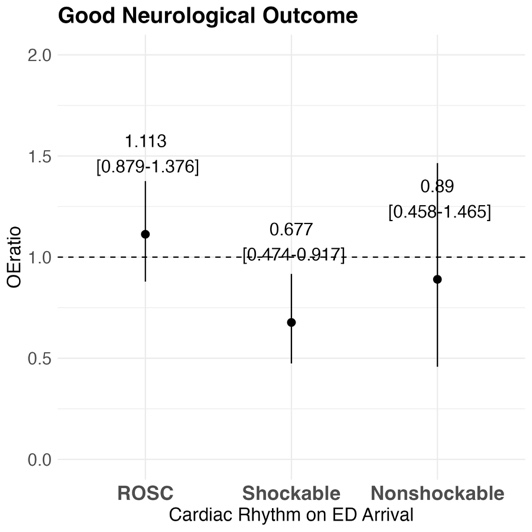

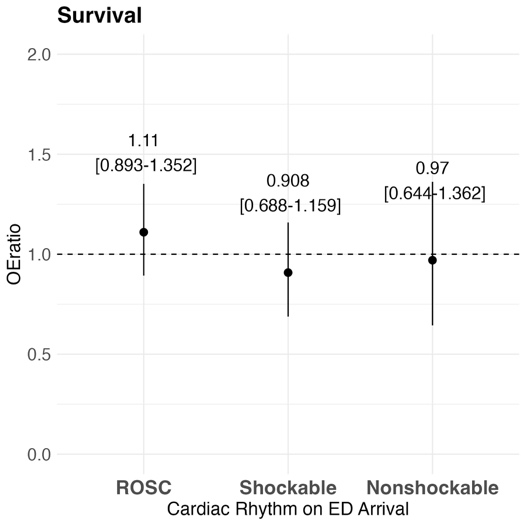

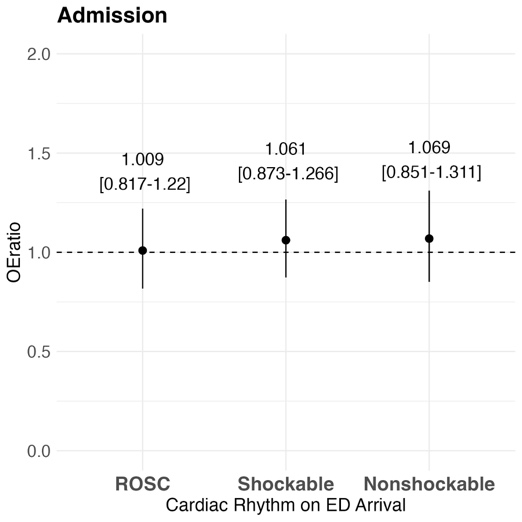


**S-Results 7. Relationship observation and prediction in the SG-PAROS**

**
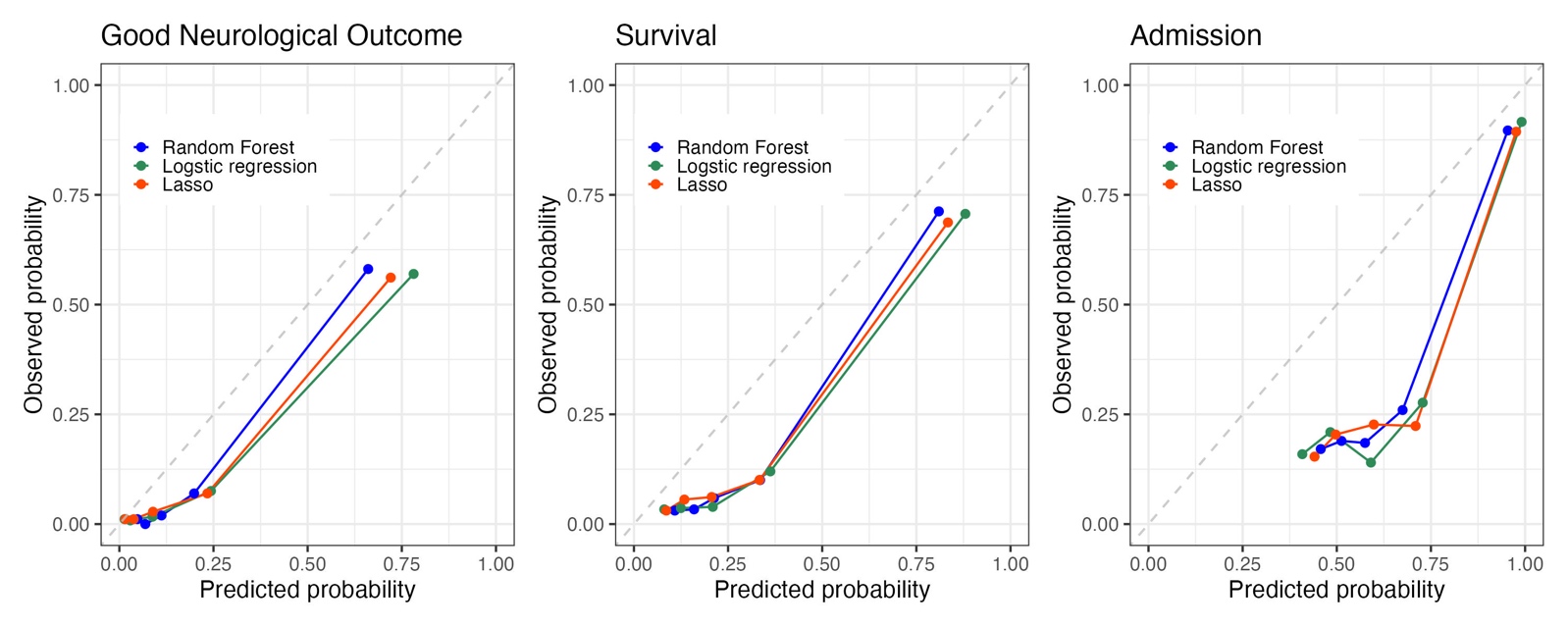
**

The diagonal line implies the perfect agreement between prediction and observation.

All the plots are lined below the diagonal line which implies the observed outcome was worse than expected.

**S-Results 8. Observation and Prediction by cardiac rhythm on ED arrival in SG-PAROS data**

**Random forest**

| **Outcome** | **Subgroup** | **Predicted** | **Observed** |
| --- | --- | --- | --- |
| Good Neurological Outcome | ROSC | 0.683 | 0.619 |
| Good Neurological Outcome | Shockable | 0.176 | 0.065 |
| Good Neurological Outcome | Nonshockable | 0.080 | 0.011 |
| Survival | ROSC | 0.830 | 0.750 |
| Survival | Shockable | 0.279 | 0.103 |
| Survival | Nonshockable | 0.177 | 0.042 |
| Admission | ROSC | 0.954 | 0.959 |
| Admission | Shockable | 0.632 | 0.211 |
| Admission | Nonshockable | 0.525 | 0.202 |

**Logistic model**

| **Outcome** | **Subgroup** | **Predicted** | **Observed** |
| --- | --- | --- | --- |
| Good Neurological Outcome | ROSC | 0.772 | 0.619 |
| Good Neurological Outcome | Shockable | 0.243 | 0.065 |
| Good Neurological Outcome | Nonshockable | 0.035 | 0.011 |
| Survival | ROSC | 0.889 | 0.750 |
| Survival | Shockable | 0.337 | 0.103 |
| Survival | Nonshockable | 0.133 | 0.042 |
| Admission | ROSC | 0.994 | 0.959 |
| Admission | Shockable | 0.690 | 0.211 |
| Admission | Nonshockable | 0.487 | 0.202 |

**Lasso**

| **Outcome** | **Subgroup** | | **Predicted** | **Observed** |
| --- | --- | --- | --- | --- |
| Good Neurological Outcome | | ROSC | 0.650 | 0.619 |
| Good Neurological Outcome | | Shockable | 0.246 | 0.065 |
| Good Neurological Outcome | | Nonshockable | 0.054 | 0.011 |
| Survival | | ROSC | 0.765 | 0.750 |
| Survival | | Shockable | 0.336 | 0.103 |
| Survival | | Nonshockable | 0.152 | 0.042 |
| Admission | | ROSC | 0.934 | 0.959 |
| Admission | | Shockable | 0.707 | 0.211 |
| Admission | | Nonshockable | 0.505 | 0.202 |

**S-Results 9. OE ratios by cardiac rhythm on ED arrival in SG-PAROS data**

**Random Forest**

See Figure 3

**Logistic model**

**
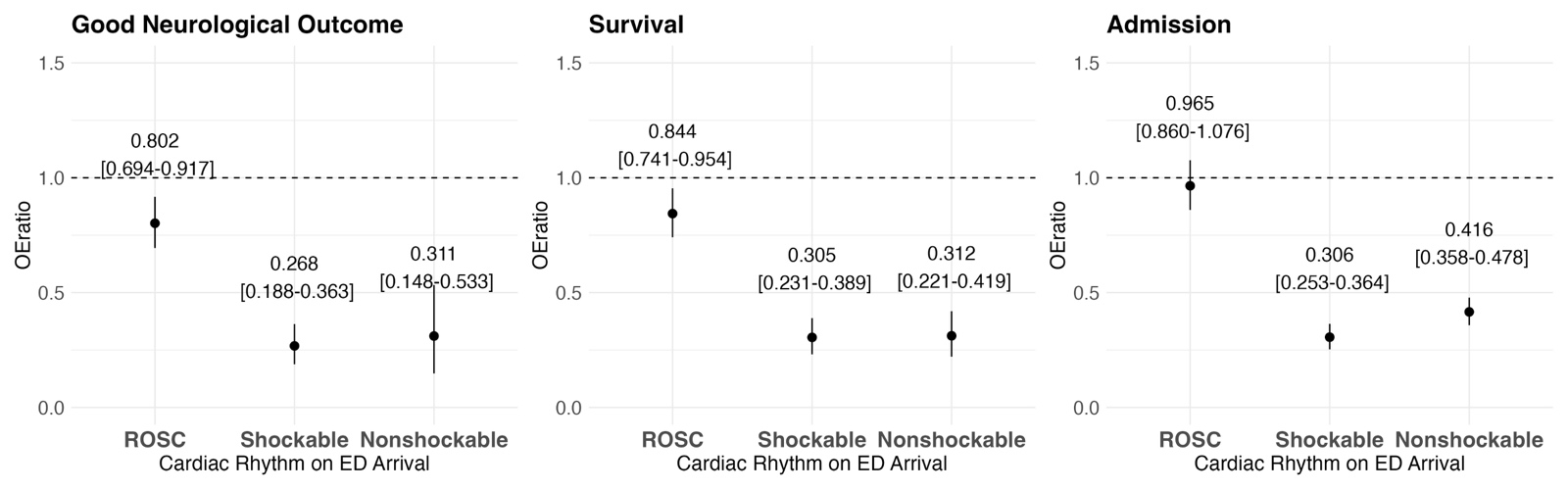
**

**Lasso**

**
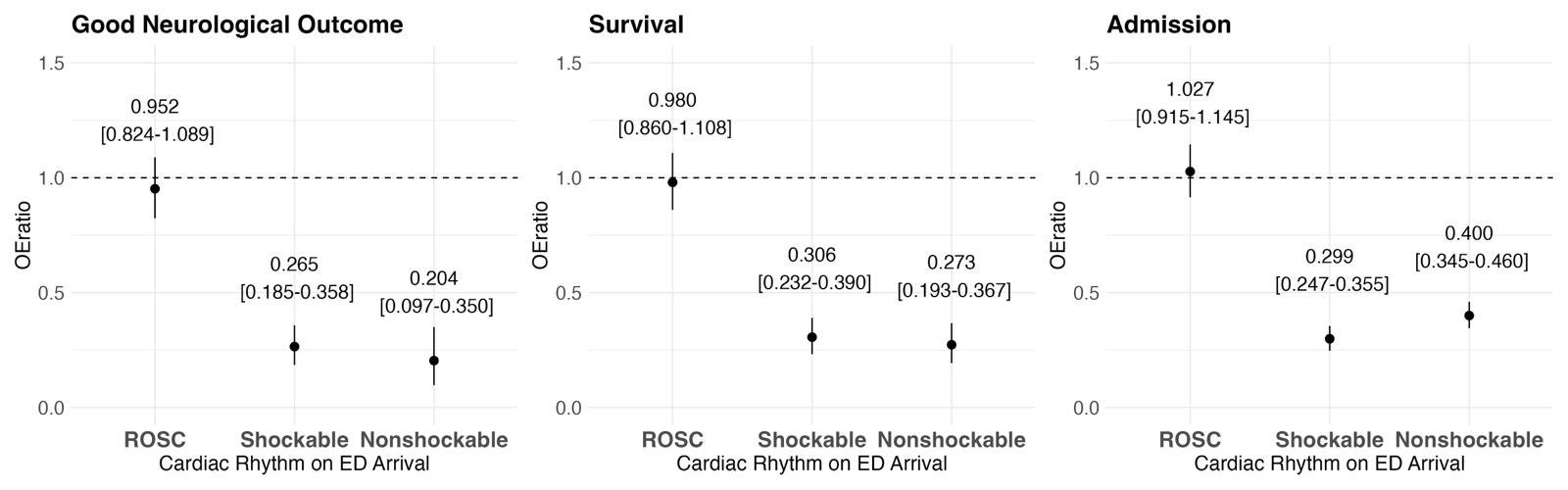
**

**S-Results 10. The results of sensitivity analysis (The model in Sensitivity analysis 2)**

We excluded the following variables in the random forest model: airway management and prehospital drug administration.

**Random Forest Model Features**

| **Predictor** | **Neurological** | **Survival** | **Admission** |
| --- | --- | --- | --- |
| Sex (Women) | 2.1 | 1 | 0.2 |
| Age | 14.2 | 5 | 3.1 |
| Witness (Yes) | 1.4 | 2.4 | 0.3 |
| Bystander CPR (Yes) | 0.8 | 2.8 | 0.7 |
| Bystander AED (Yes) | 0 | 0 | 0 |
| Prehospital ROSC (Yes) | 97.6 | 100 | 100 |
| Time to ED arrival | 10.9 | 10 | 7.4 |
| Cardiac rhythm on ED arrival (ROSC) | 100 | 85.3 | 61.1 |
| Cardiac rhythm on ED arrival (Shockable) | 15.7 | 12.9 | 21.4 |

CPR, Cardiopulmonary resuscitation, Shockable: Ventricular fibrillation and pulseless ventricular tachycardia, ROSC, Return of spontaneous circulation, AED, Automated external defibrillator, ROSC, Return of spontaneous circulation, ED: Emergency department.

| **Outcome** | **Model** | **C-stat** | **R2** | **Brier** | **Intercept** | **Slope** |
| --- | --- | --- | --- | --- | --- | --- |
| Good Neurological Outcome | Random Forest | 0.877 | 0.503 | 0.133 | -0.055 | 1.08 |
| Survival | Random Forest | 0.854 | 0.462 | 0.158 | 0.172 | 1.016 |
| Admission | Random Forest | 0.793 | 0.238 | 0.142 | 0.372 | 0.912 |

C-stat: C-statistics

**ROC (Receiver operating curve) and Calibration plot in the validation dataset**

**
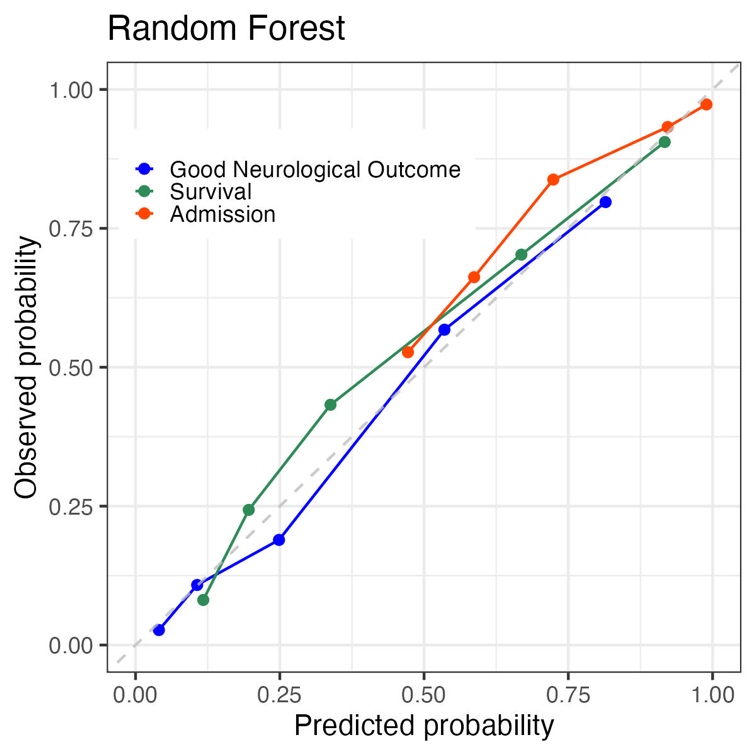
**

**S-Results 11. The results of sensitivity analysis (Outcomes in Sensitivity analysis 2)**

**Relationship between observation and prediction in the SG-PAROS data**

**
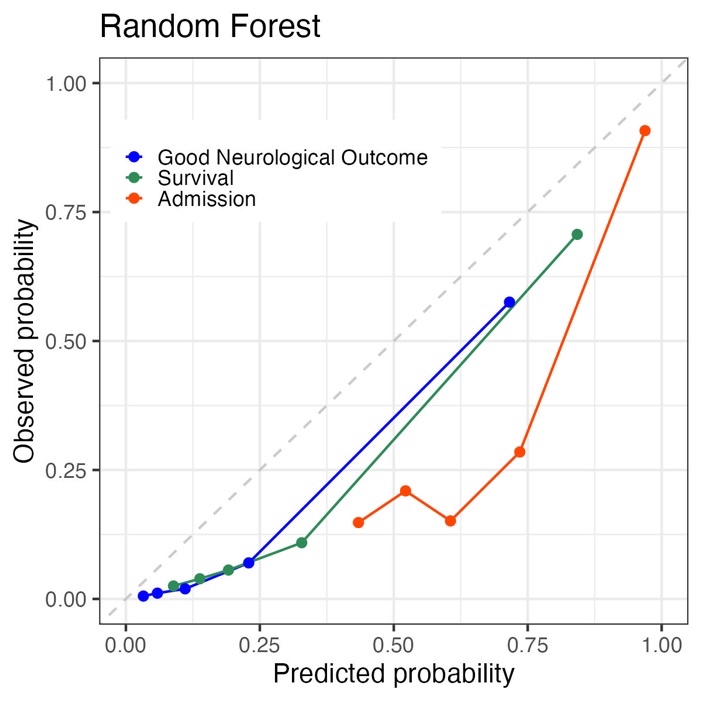
**

The diagonal line implies the perfect agreement between prediction and observation.

All the plots are lined below the diagonal line which implies the observed outcome was worse than expected.

**The OE ratio by the cardiac rhythm on ED arrival in SG-PAROS data**

**
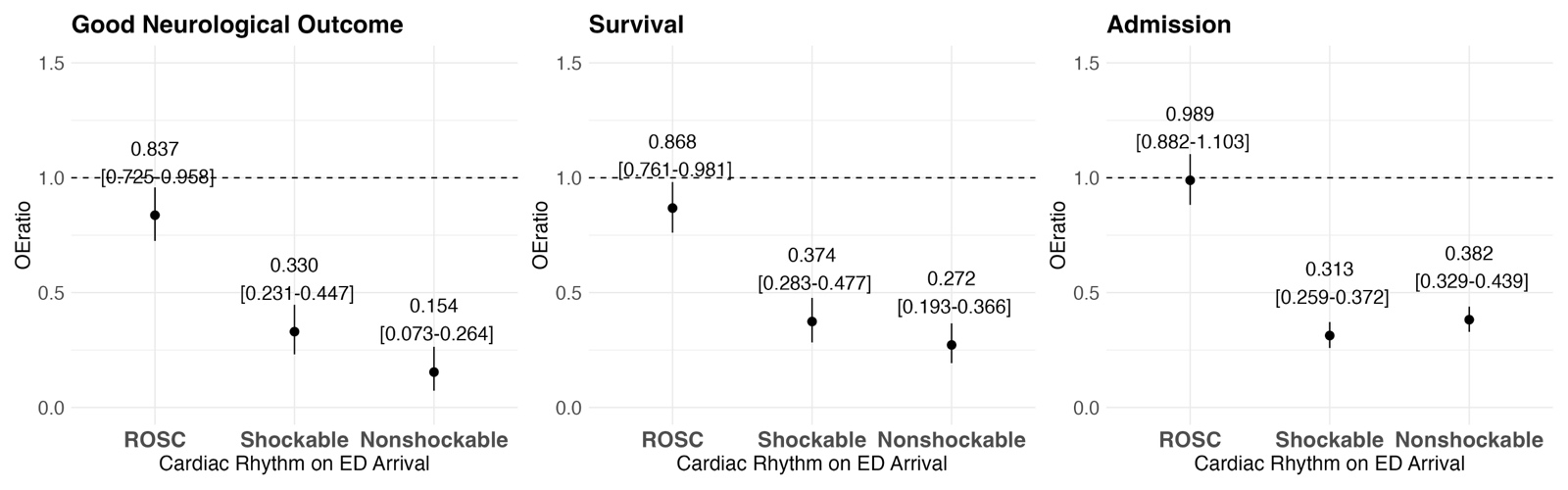
**

The OE ratios for the outcomes among the patients with ROSC on ED arrival are around 1.0, implying that the outcomes in SG-PAROS are the same level as those of Osaka-CRITICAL 2012-2017. On the other hand, The OE ratios for the outcomes among the patients without ROSC on ED arrival (shockable or nonshockable) are substantially lower than 1.0, implying that the outcomes in SG-PAROS are worse than those of Osaka-CRITICAL 2012-2017.

**S- Results 12. The results of sensitivity analysis 3 (Characteristics)**

**Patient characteristics of SG-PAROS**

| Characteristic | 2010-2016, N = 1,789 | 2017-2020, N = 1,525 |
| --- | --- | --- |
| Men | 1,546 (86%) | 1,324 (87%) |
| Age (years) | 57 (50, 65) | 59 (50, 65) |
| Witness | 1,373 (77%) | 1,157 (76%) |
| Bystander CPR | 988 (55%) | 1,090 (71%) |
| Bystander AED | 143 (8.0%) | 320 (21%) |
| Prehospital Airway |  |  |
| Intubation | 11 (0.6%) | 6 (0.4%) |
| Supraglottic Airway | 1,557 (87%) | 1,303 (85%) |
| None | 221 (12%) | 216 (14%) |
| Prehospital Drug | 1,072 (60%) | 1,407 (92%) |
| Prehospital ROSC | 365 (20%) | 537 (35%) |
| Time to ED arrival (min) | 35 (30, 40) | 37 (33, 43) |
| In-hospital procedures |  |  |
| ECMO | 10 (0.6%) | 11 (0.7%) |
| PCI | 309 (17%) | 344 (23%) |
| TTM | 203 (11%) | 329 (22%) |
| Outcome |  |  |
| Admission | 596 (33%) | 598 (39%) |
| Survival | 335 (19%) | 393 (26%) |
| Good Neurological Outcome | 244 (14%) | 336 (22%) |

Continuous variables are median and interquartile range, and categorical variables are number and percentage (%).

CPR, Cardiopulmonary resuscitation, AED, Automated external defibrillator, ROSC, Return of spontaneous circulation,

ED, Emergency department, Shockable: Ventricular fibrillation and pulseless ventricular tachycardia, Unshockable: Pulseless electrical activity and asystole, ECMO, Extracorporeal membrane oxygenation, PCI, Percutaneous coronary intervention, TTM, Targeted temperature management

**S-Results 13. The results of sensitivity analysis 3 (Model in Sensitivity analysis 3)**

We developed the random forest model in which we excluded the cardiac rhythm on ED arrival for predicting the outcomes using the Osaka-CRITICAL data 2012-2017. The model validation was evaluated using the Osaka-CRITICAL data 2018-2019. Then, we calculated the OE ratios of SG-PAROS 2017-2020 using the developed models.

**Random Forest Model Features**

| **Predictor** | **Neurological** | **Survival** | **Admission** |
| --- | --- | --- | --- |
| Sex (Women) | 1.6 | 0.9 | 0 |
| Age | 5.1 | 2.1 | 3.2 |
| Witness (Yes) | 1.8 | 1.5 | 18.5 |
| Bystander CPR (Yes) | 1.5 | 1 | 12.5 |
| Bystander AED (Yes) | 0 | 0 | 5.7 |
| Pre-hospital airway (None) | 20.6 | 11.4 | 43.8 |
| Pre-hospital airway (Supraglottic airway) | 5 | 5.4 | 7.6 |
| Pre-hospital drug administration (Yes) | 14.2 | 10.8 | 14.1 |
| Prehospital ROSC (Yes) | 100 | 100 | 100 |
| Time to ED arrival | 4.5 | 4.6 | 25.6 |

CPR, Cardiopulmonary resuscitation, Shockable: Ventricular fibrillation and pulseless ventricular tachycardia, ROSC, Return of spontaneous circulation, AED, Automated external defibrillator, ROSC, Return of spontaneous circulation, ED: Emergency department.

| **Outcome** | **Model** | **C-stat** | **R2** | **Brier** | **Intercept** | **Slope** |
| --- | --- | --- | --- | --- | --- | --- |
| Good Neurological Outcome | Random Forest | 0.882 | 0.508 | 0.126 | -0.041 | 1.426 |
| Survival | Random Forest | 0.813 | 0.391 | 0.169 | 0.111 | 1.099 |
| Admission | Random Forest | 0.691 | -0.258 | 0.173 | 0.784 | 0.271 |

C-stat: C-statistics

**ROC (Receiver operating curve) and Calibration plot in the validation dataset**


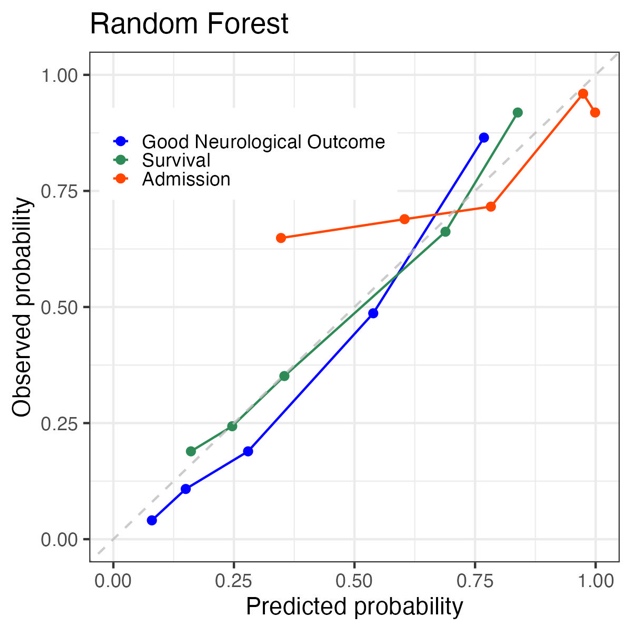


**S-Results 14. The results of sensitivity analysis 3 (Outcomes in Sensitivity analysis 3)**

**Relationship between observation and prediction in the SG-PAROS data**


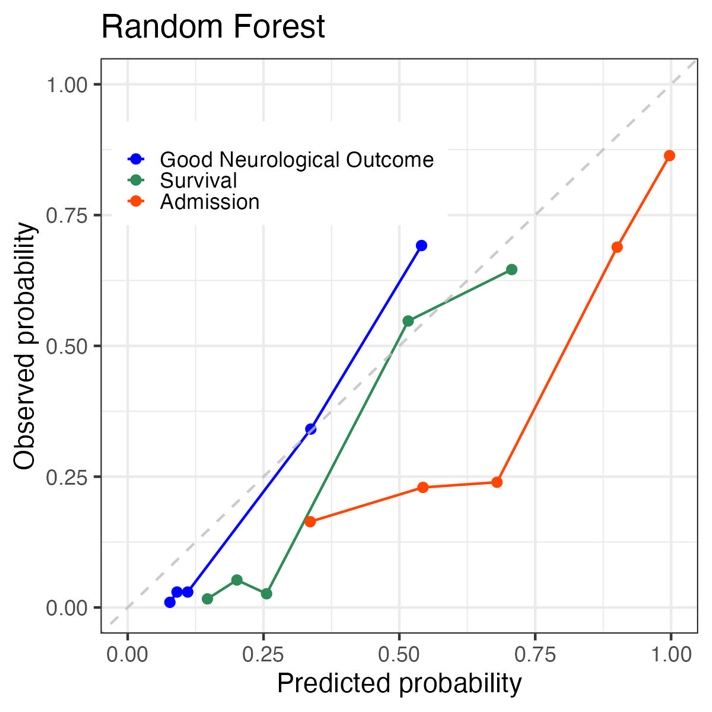


**The OE ratio by the cardiac rhythm on ED arrival in SG-PAROS data**


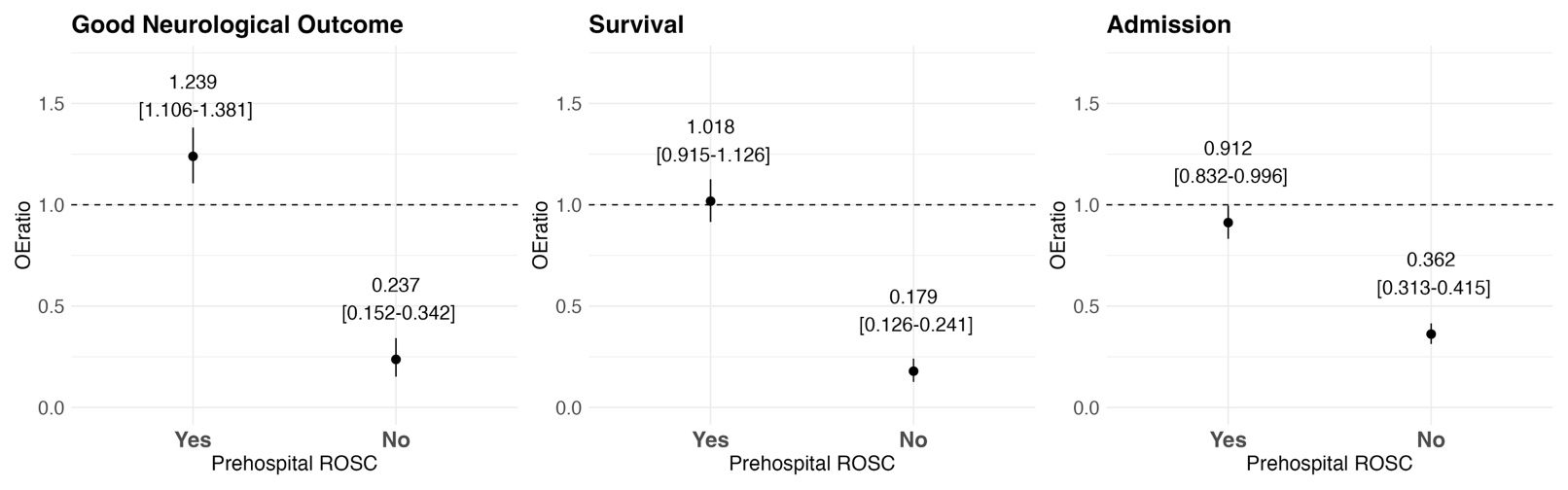


The OE ratios for the outcomes among the patients with prehospital ROSC are around 1.0, implying that the outcomes in SG-PAROS 2017-2020 are the same level as those of Osaka-CRITICAL 2012-2017. On the other hand, The OE ratios for the outcomes among the patients without prehospital ROSC are substantially lower than 1.0, implying that the outcomes in SG-PAROS 2017-2020 are worse than those of Osaka-CRITICAL 2012-2017.

**Reference**

1. Kitamura T, Iwami T, Kawamura T, Nagao K, Tanaka H, Hiraide A. Nationwide public-access defibrillation in Japan. *N Engl J Med*. 2010 Massachusetts Medical Society; 2010:994-1004. vol. 11.

2. Kitamura T, Kiyohara K, Sakai T, et al. Public-Access Defibrillation and Out-of-Hospital Cardiac Arrest in Japan. *N Engl J Med*. Oct 27 2016;375(17):1649-1659. doi:10.1056/NEJMsa1600011

3. Jacobs I, Nadkarni V, Bahr J, et al. Cardiac arrest and cardiopulmonary resuscitation outcome reports: update and simplification of the Utstein templates for resuscitation registries: a statement for healthcare professionals from a task force of the International Liaison Committee on Resuscitation (American Heart Association, European Resuscitation Council, Australian Resuscitation Council, New Zealand Resuscitation Council, Heart and Stroke Foundation of Canada, InterAmerican Heart Foundation, Resuscitation Councils of Southern Africa). *Circulation*. 2004:3385-97. vol. 21.

4. Yamada T, Kitamura T, Hayakawa K, et al. Rationale, design, and profile of Comprehensive Registry of In-Hospital Intensive Care for OHCA Survival (CRITICAL) study in Osaka, Japan. *J Intensive Care*. 2016;4:10. doi:10.1186/s40560-016-0128-5

5. Communications MoIAa. Statistics Bureau. Accessed Nov, 17th, 2019. <https://www.stat.go.jp/english/>

6. Fire and Disaster Management Agency of the Ministry of Internal Affairs and Communications  website. Accessed 7, June

 2018. <http://www.fdma.go.jp/neuter/topics/fieldList9_3.html>

7. Ong ME, Shin SD, Tanaka H, et al. Pan-Asian Resuscitation Outcomes Study (PAROS): rationale, methodology, and implementation. *Acad Emerg Med*. Aug 2011;18(8):890-7. doi:10.1111/j.1553-2712.2011.01132.x

8. Sakamoto T, Morimura N, Nagao K, et al. Extracorporeal cardiopulmonary resuscitation versus conventional cardiopulmonary resuscitation in adults with out-of-hospital cardiac arrest: a prospective observational study. *Resuscitation*. Jun 2014;85(6):762-8. doi:10.1016/j.resuscitation.2014.01.031

9. Sakamoto T, Asai Y, Nagao K, et al. Multicenter non-randomized prospective cohort study of extracorporeal cardiopulmonary resuscitation for out-of hospital cardiac arrest: Study of Advanced Life Support for Ventricular Fibrillation with Extracorporeal Circulation in Japan (SAVE-J). Am Heart Assoc; 2011.

10. Inoue A, Hifumi T, Sakamoto T, et al. Extracorporeal cardiopulmonary resuscitation in adult patients with out-of-hospital cardiac arrest: a retrospective large cohort multicenter study in Japan. *Crit Care*. May 9 2022;26(1):129. doi:10.1186/s13054-022-03998-y

11. Hifumi T, Inoue A, Takiguchi T, et al. Variability of extracorporeal cardiopulmonary resuscitation practice in patients with out-of-hospital cardiac arrest from the emergency department to intensive care unit in Japan. *Acute Medicine & Surgery*. 2021;8(1):e647. doi:<https://doi.org/10.1002/ams2.647>

12. Waljee AK, Mukherjee A, Singal AG, et al. Comparison of imputation methods for missing laboratory data in medicine. *BMJ Open*. 2013;3(8):e002847. doi:10.1136/bmjopen-2013-002847

13. Stekhoven DJ, Bühlmann P. MissForest—non-parametric missing value imputation for mixed-type data. *Bioinformatics*. 2012;28(1):112-118. doi:10.1093/bioinformatics/btr597

14. Moons KG, Altman DG, Reitsma JB, et al. Transparent Reporting of a multivariable prediction model for Individual Prognosis or Diagnosis (TRIPOD): explanation and elaboration. *Ann Intern Med*. Jan 6 2015;162(1):W1-73. doi:10.7326/m14-0698

15. Steyerberg EW, Harrell FE, Jr. Prediction models need appropriate internal, internal-external, and external validation. *J Clin Epidemiol*. Jan 2016;69:245-7. doi:10.1016/j.jclinepi.2015.04.005

16. Collins GS, Ogundimu EO, Altman DG. Sample size considerations for the external validation of a multivariable prognostic model: a resampling study. *Statistics in medicine*. 2016;35(2):214-226. doi:10.1002/sim.6787

17. Goto T, Camargo CA, Jr., Faridi MK, Freishtat RJ, Hasegawa K. Machine Learning–Based Prediction of Clinical Outcomes for Children During Emergency Department Triage. *JAMA Network Open*. 2019;2(1):e186937-e186937. doi:10.1001/jamanetworkopen.2018.6937

18. Parikh RB, Manz C, Chivers C, et al. Machine Learning Approaches to Predict 6-Month Mortality Among Patients With Cancer. *JAMA Network Open*. 2019;2(10):e1915997-e1915997. doi:10.1001/jamanetworkopen.2019.15997

19. Patel SJ, Chamberlain DB, Chamberlain JM. A Machine Learning Approach to Predicting Need for Hospitalization for Pediatric Asthma Exacerbation at the Time of Emergency Department Triage. *Acad Emerg Med*. Dec 2018;25(12):1463-1470. doi:10.1111/acem.13655

20. Levin S, Toerper M, Hamrock E, et al. Machine-Learning-Based Electronic Triage More Accurately Differentiates Patients With Respect to Clinical Outcomes Compared With the Emergency Severity Index. *Ann Emerg Med*. May 2018;71(5):565-574.e2. doi:10.1016/j.annemergmed.2017.08.005

21. Kuhn M, Johnson K, service S. *Applied Predictive Modeling*. Springer New York : Imprint: Springer; 2013.

22. Package ‘ranger’. Accessed Oct, 22th, 2020. <https://cran.r-project.org/web/packages/ranger/ranger.pdf>

23. Package ‘caret’. Accessed Oct, 22th, 2020. <https://cran.r-project.org/web/packages/caret/caret.pdf>

24. Package ‘xgboost’. Accessed Oct, 22th, 2020. <https://cran.r-project.org/web/packages/xgboost/xgboost.pdf>

25. Desai RJ, Wang SV, Vaduganathan M, Evers T, Schneeweiss S. Comparison of Machine Learning Methods With Traditional Models for Use of Administrative Claims With Electronic Medical Records to Predict Heart Failure Outcomes. *JAMA Network Open*. 2020;3(1):e1918962-e1918962. doi:10.1001/jamanetworkopen.2019.18962

26. Frizzell JD, Liang L, Schulte PJ, et al. Prediction of 30-Day All-Cause Readmissions in Patients Hospitalized for Heart Failure: Comparison of Machine Learning and Other Statistical Approaches. *JAMA Cardiology*. 2017;2(2):204-209. doi:10.1001/jamacardio.2016.3956

27. Liang W, Liang H, Ou L, et al. Development and Validation of a Clinical Risk Score to Predict the Occurrence of Critical Illness in Hospitalized Patients With COVID-19. *JAMA Internal Medicine*. 2020;180(8):1081-1089. doi:10.1001/jamainternmed.2020.2033

28. Delahanty RJ, Alvarez J, Flynn LM, Sherwin RL, Jones SS. Development and Evaluation of a Machine Learning Model for the Early Identification of Patients at Risk for Sepsis. *Ann Emerg Med*. Apr 2019;73(4):334-344. doi:10.1016/j.annemergmed.2018.11.036

29. Raita Y, Goto T, Faridi MK, Brown DFM, Camargo CA, Jr., Hasegawa K. Emergency department triage prediction of clinical outcomes using machine learning models. *Crit Care*. Feb 22 2019;23(1):64. doi:10.1186/s13054-019-2351-7

30. Sidey-Gibbons JAM, Sidey-Gibbons CJ. Machine learning in medicine: a practical introduction. *BMC Medical Research Methodology*. 2019/03/19 2019;19(1):64. doi:10.1186/s12874-019-0681-4

31. Tibshirani R. Regression Shrinkage and Selection via the Lasso. *Journal of the Royal Statistical Society Series B (Methodological)*. 1996;58(1):267-288.

32. Pavlou M, Ambler G, Seaman SR, et al. How to develop a more accurate risk prediction model when there are few events. *BMJ : British Medical Journal*. 2015;351:h3868. doi:10.1136/bmj.h3868

33. Package ‘glmnet’. Accessed Oct, 22th, 2020. <https://cran.r-project.org/web/packages/glmnet/glmnet.pdf>
